# Multifunctional N-P-doped carbon dots for regulation of apoptosis and autophagy in B16F10 melanoma cancer cells and *in vitro* imaging applications

**DOI:** 10.7150/thno.42291

**Published:** 2020-06-22

**Authors:** Vivek K. Bajpai, Imran Khan, Shruti Shukla, Sung-Min Kang, Faisal Aziz, Kumud Malika Tripathi, Deepika Saini, Hye-Jin Cho, Nam Su Heo, Sumit K. Sonkar, Lei Chen, Yun Suk Huh, Young-Kyu Han

**Affiliations:** 1 Department of Energy and Materials Engineering, Dongguk University-Seoul, 30 Pildong-ro 1-gil, Seoul 04620, Republic of Korea.; 2 Department of Biological Engineering, Biohybrid Systems Research Center (BSRC), Inha University, 100 Inha-ro, Nam-gu, Incheon 22212, Republic of Korea.; 3 The Hormel Institute, University of Minnesota, Austin, MN, 55912, USA.; 4 Department of Food Science and Technology, National Institute of Food Technology Entrepreneurship and Management (NIFTEM), Sonipat, Haryana 131028, India.; 5 Wallace H. Coulter Department of Biomedical Engineering, Georgia Institute of Technology and Emory School of Medicine, Atlanta, Georgia, 30332, USA.; 6 Department of Chemistry, Indian Institute of Petroleum and Energy, Visakhapatnam 531035, Andhra Pradesh, India.; 7 Department of Chemistry, Malaviya National Institute of Technology, Jaipur, Jaipur 302017, India.; 8 Reliability Assessment Center for Chemical Materials, Korea Research Institute of Chemical Technology (KRICT), 141 Gajeong-ro, Yuseong-gu, Daejeon 305-600, Republic of Korea.; 9 Research Center for Materials Analysis, Korea Basic Science Institute (KBSI), Daejeon 34133, Republic of Korea.; 10 College of Food Science, Fujian Agriculture and Forestry University, Fuzhou, Fujian 350002, China.

**Keywords:** N-P-doping, apoptosis, autophagy, bioimaging, cell cycle arrest, B16F10 melanoma cells

## Abstract

**Rationale:** The present study reports the multifunctional anticancer activity against B16F10 melanoma cancer cells and the bioimaging ability of fluorescent nitrogen-phosphorous-doped carbon dots (NPCDs).

**Methods:** The NPCDs were synthesized using a single-step, thermal treatment and were characterized by TEM, XPS, fluorescence and UV-Vis spectroscopy, and FTIR analysis. The anticancer efficacy of NPCDs was confirmed by using cell viability assay, morphological evaluation, fluorescent live-dead cell assay, mitochondrial potential assay, ROS production, RT-PCR, western-blot analysis, siRNA transfection, and cellular bioimaging ability.

**Results:** The NPCDs inhibited the proliferation of B16F10 melanoma cancer cells after 24 h of treatment and induced apoptosis, as confirmed by the presence of fragmented nuclei, reduced mitochondrial membrane potential, and elevated levels of reactive oxygen species. The NPCDs treatment further elevated the levels of pro-apoptotic factors and down-regulated the level of Bcl2 (B-cell lymphoma 2) that weakened the mitochondrial membrane, and activated proteases such as caspases. Treatment with NPCDs also resulted in dose-dependent cell cycle arrest, as indicated by reduced cyclin-dependent kinase (CDK)-2, -4, and -6 protein levels and an enhanced level of p21. More importantly, the NPCDs induced the activation of autophagy by upregulating the protein expression levels of LC3-II and ATG-5 (autophagy-related-5) and by downregulating p62 level, validated by knockdown of ATG-5. Additionally, owing to their excellent luminescence property, these NPCDs were also applicable in cellular bioimaging, as evidenced by the microscopic fluorescence imaging of B16F10 melanoma cells.

**Conclusion:** Based on these findings, we conclude that our newly synthesized NPCDs induced cell cycle arrest, autophagy, and apoptosis in B16F10 melanoma cells and presented good cellular bioimaging capability.

## Introduction

Melanoma is a highly resistant and malignant cancer accounting for around 3% of all malignant tumor cases, and its incidence has increased faster than any other cancer type during recent decades. Despite accounting for only 4% of all skin cancer cases, it is the most aggressive and causes approximately 80% of all deaths from skin cancer 1. If diagnosed early in the course of the disease, melanoma is readily cured by simple wide surgical excision 1. However, once it metastasizes, there is presently no treatment that reliably affects the course of the disease. Hence, there is a medical necessity for new nanodrugs that have the potential of inducing apoptosis in cancer cells but with no adverse side effects in healthy cells 2.

There are various pathways for cancer cell death, such as apoptosis, autophagy, or necrosis. Apoptosis is programmed cell death during which reactive oxygen species (ROS) are free radicals generated in response to external stimuli 3. The generated ROS causes stress on the mitochondria, leading to dysregulation of the mitochondrial membrane and protein markers, and the released cytochrome C causes activation of proteases such as caspases, which cause apoptotic cell death 3-5.

In addition to apoptosis, macroautophagy (autophagy) is a well-known conserved orchestrated process for removing damaged cellular organelles or unused metabolites 6. Moreover, autophagy is a lysosomal catabolic process that is very well known for its dual function as cell survival and cell death. During starvation, the autophagic process provides biofuel to damaged organelles, which further affects cell survival or cell death in a context-dependent manner. The autophagic process can be activated by external stimuli, which also induces caspase-independent cell death 6. Furthermore, necrosis is characterized morphologically by widespread swelling of cell membranes, often escorted by chromatin condensation and an uneven DNA degradation pattern. The release of the intracellular contents leads to enormous cellular damage that affects neighboring cells, which elucidates why necrosis triggers inflammatory and autoimmune reactions 7. Several treatment strategies have been applied to treat a variety of cancers, but they have shown less effectiveness. Therefore, it is essential to find nanodrugs as alternatives to chemical drugs to eradicate cancer cells 8.

Considering similar applications, presently, the fluorescence imaging of cells, biological tissues, and specific localized sub-cellular organelles (nuclei, mitochondria, and/or lysosomes) based on fluorescent molecules have attracted considerable attention across medical fields for an in-depth understanding of biological processes and pathways toward early disease diagnosis and drug development in biomedicine 9-11. Besides, fluorescence imaging can visualize the expression and activity of specific molecules as well as cellular and biological processes that influence tumor behavior and response to therapeutic drugs 12. For example, the nucleolus is the key site in the nucleus that synthesizes, processes, and assembles the ribosomal ribonucleic acids 13, and its function is closely related to cell growth and proliferation 13. Enlarged or aberrant nucleoli and their associated number are often indicative of types of cancer and other human disorders 13. In the case of mitochondria and lysosomes, permeabilization of their constitutes is one of the major checkpoints in inducing apoptotic and necrotic cell death; both of them play pivotal roles in cell life and death 13.

For the imaging purposes, multi-emissive carbon dots (CDs) 13, is a well-known example of nanotechnology, that have attracted significant attention in the field of the imaging applications due to their unique core structure with rich surface functional species 14. Now, the optical and electronic properties of CD can be enhanced via the process of doping 15-17, that can either in the form of metal ions and heteroatom (e.g. with N, B, P etc.), individually 18, 19 or multiple 20-22 and sometimes its self-doping 23, 24. Doping of N and P considerably improves the chemical activity and physicochemical properties of CDs due to the generation of the new surficial active sites in terms of surface defects that can improve their optical properties 25. Along with these, they also possess excellent photochemical stability, resistance to photo-bleaching, and excellent biocompatibility. That makes them useful in various other fields as photocatalysis 26, sensing 18, 24, electrochemistry 27, and biological imaging 28-31.

In this article, a simple high-yield synthetic protocol for the fabrications of multi-heteroatom (N-P) co-doped CDs (NPCDs) using a single-step, thermal treatment is reported, where imidazole is used as the N source, phosphoric acid as the P source, and polyethylene glycol (PEG) as the carbon precursor. The as-synthesized NPCDs showed a good value of quantum yield ~9%. Also, we explored the underlying mechanism of the NPCDs' anticancer potential against B16F10 melanoma cells and their cellular bioimaging application* in vitro*.

## Materials and Methods

### Chemicals and reagents

The chemical reagents such as PEG, ammonia solution, imidazole, and phosphoric acid were procured from Sigma-Aldrich, India. Reagents for microscopy analysis like osmium tetroxide, glutaraldehyde, and ethanol were procured from Duksan Chemicals (Gyeonggi-do, South Korea). All other chemicals used were of analytical grade and supplied by Sigma-Aldrich (St. Louis, MO, USA), and all the synthesis experiments were executed in deionized (DI) water.

### Preparation of the NPCDs

**Scheme 1:**
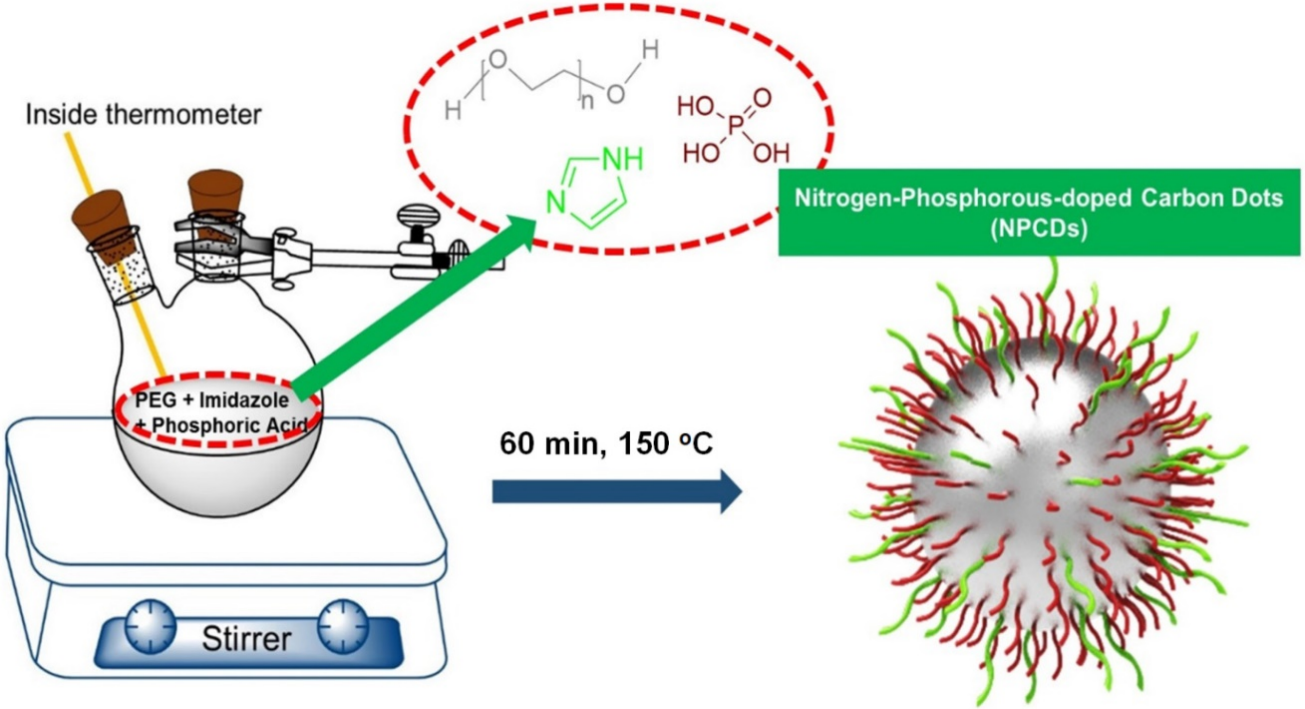
Schematic representation showing the synthesis of nitrogen-phosphorous-doped carbon dots (NPCDs).

The preparation of NPCDs was done by the heating process of reagents 3 g imidazole (N source), 10 mL PEG (carbon source), and 10 mL phosphoric acid (P source) in a two neck round bottom flask by maintaining the temperature at 150 °C for 60 min. 28, 32, 33 The excess of phosphoric acid was removed by the addition of ammonia solution in the as-prepared brown solution followed by the centrifugation process (30 min at 6,000 rpm, three repetitive steps) and dried resultant supernatant for removal of excess water. Afterward, the produced solution was termed as NPCDs from Scheme 1. The QY of NPCDs was ~ 9 % (slightly different for different batches) at 348 nm wavelength, calculated by the given formula:


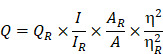


Where QY is represented as Q, the measured integrated emission intensity as I, refractive index of the solvent as η and A is the optical density. The subscript “R” refers to the reference standard of quinine sulphate with known QY 28, 34, 35.

### Instrumentation used for the characterization of NPCDs

NPCDs examination: Transmission electron microscopy (TEM; JEM-2100F, JEOL, Japan) at 200 kV was used for internal morphology analysis. X-ray photoelectron spectroscopy (XPS) was applied for dopant and surficial group analysis using ESCA system (Omicron Nanotechnology, Oxford Instruments, Malaviya National Institute of Technology Jaipur (MNIT), India). Fluorescence measurements were done using a Fluoromax 4C-L system (Horiba Scientific, NJ, USA and Fluoromax 4C.L. Spectrophotometer, MNIT, India). Jasco V-730 spectrophotometer, MNIT, India recorded ultraviolet-visible (UV-Vis) spectra, whereas a Bruker Fourier-transform infrared (FTIR) spectrometer (Vertex 70 model, MNIT, India) was used for the functional group analysis.

### Cell lines

B16F10 melanoma cells, colon cancer cells (HCT 116 and HT29), lung cancer cells (A549) and cervical cancer cells (HeLa), BEAS-2B human lung epithelial cells, and HACAT human keratinocytes were purchased from the American Type Culture Collection (Manassas, VA, USA). These cells were cultured in RPMI-1640 (Invitrogen, Carlsbad, CA, USA) media, supplemented with 10% (v/v) fetal calf serum (Invitrogen, Carlsbad, CA, USA) and a 1% penicillin-streptomycin cocktail at 37 °C in a 5% CO_2_ incubator 36.

### Cell viability, cytotoxicity and morphological evaluation

The cytotoxic effect of NPCDs was first determined by 3-(4,5-dimethylthiazol-2-yl)-2,5-diphenyl tetrazolium bromide (MTT) assay 37. Briefly, 5×10^4^ cells/well of various cancer cells such as B16F10 melanoma cells, colon cancer cells (HCT 116 and HT29), lung cancer cells (A549) and cervical cancer cells (HeLa), BEAS-2B human lung epithelial cells, HACAT human keratinocytes were seeded and after attachment further incubated for 24 h in the presence of various concentrations of NPCDs (0-120 μL/mL) at 37 °C in a CO_2_ incubator. After incubation, the cells were treated with MTT solution (5 mg/mL), yielding dark-blue-colored formazan crystals, which were dissolved in 50 μL dimethyl sulfoxide (DMSO). The absorbance (540 nm) of these crystals was measured using a microplate reader (Bio-Tek instrument Co., WA, USA).

Cytotoxicity was further confirmed by the lactate dehydrogenase (LDH) assay. Briefly, cells were treated with various concentrations of NPCDs for 24 h at 37 °C. After incubation, media were removed and processed to evaluate extracellular LDH release using an LDH detection kit (Sigma-Aldrich, St. Louis, MO, USA) as per the manufacturer's guidelines. Cytotoxicity was evaluated by calculating the absorbance at 490 and 690 nm.

In addition, a microscopic examination was carried out to determine the morphological changes in B16F10 cells after exposure to NPCDs using an inverted microscope (Nikon Eclipse TS200, Nikon Corp., Tokyo, Japan).

Cells exhibiting apoptotic characteristics are sensitive to formamide. Therefore, denatured DNA was detected using a monoclonal antibody against single-stranded DNA with an ApoStrand™ ELISA (enzyme-linked immunosorbent assay) apoptosis detection kit (Enzo Life Sciences, Plymouth Meeting, PA, USA) in accordance with the manufacturer's instructions.

During the clonogenic assay, cells were seeded in a 12-well culture plate, allowed to attach for 24 h, and then subjected to treatment with NPCDs at different concentrations. After seven days of incubation at 37 °C in a CO_2_ incubator, cells were washed with phosphate buffer saline (PBS) and stained with 0.5% crystal violet 38.

### Scanning electron microscopy (SEM) and Bio-transmission electron microscopy (bio-TEM) studies

#### Observing NPCDs-induced B16F10 morphological changes

For SEM, in brief, cells were grown on coverslips, washed with 1× PBS, and fixed with 2.5% glutaraldehyde, followed by dehydration using gradient ethanol. The coverslips were then dried in a desiccator, placed on the SEM stage, super-coated with gold, and subsequently visualized under SEM.

To observe the apoptosis morphology of B16F10 cells after transfection with nano-complexes, the NPCD-treated B16F10 cells were collected and fixed, then frozen and made into sections. The cell sections were stuck on the copper online surface and observed with TEM.

### Fluorescence staining

#### Apoptotic and live-dead morphological evaluation

Nuclear fragmentation as a marker of apoptosis was evaluated by means of Hoechst-propidium iodide (Hoechst-PI) staining. Briefly, cells (5×10^4^ cells/well) were seeded in a 24-well plate and allowed to attach for 24 h. The cells were then subjected to 24-h treatment with NPCDs (0-30 μL/mL). After incubation, the cells were stained with Hoechst-PI (1 mg/mL) and images were captured with epifluorescence imaging on an inverted microscope (Nikon Eclipse TS200, Nikon Corp., Japan). After the compound treatment, the cells were stained with Live/Dead Cell Viability Assay kit reagents (Thermo Fisher Scientific, Waltham, MA, USA) and images were captured under the aforementioned fluorescence microscope. The number of apoptotic cells and cell index were calculated as percent apoptotic nuclei compared with the total number of cells 36.

#### Oxidative stress staining

Intracellular ROS production in response to NPCD exposure of the B16F10 cells was determined using 2',7'-dichlorodihydrofluorescein diacetate (H_2_DCFDA) fluorescence stain. Briefly, cells (5×10^4^ cells/well) were seeded in a 24-well culture plate, allowed to attach for 24 h, and then subjected to treatment at different concentrations (0-30 μL/mL) of NPCDs. Afterward, the cells were washed with PBS and subsequently incubated with 20 mM H_2_DCFDA dye for 30 min 37. The cells were washed twice with PBS and ROS formation was immediately detected via epifluorescence imaging. Fluorescence intensity was quantified by using the ImageJ software (Maryland, USA).

#### Mitochondrial membrane potential (∆ψm)

As previously mentioned, B16F10 cells were grown in a 24-well culture plate and treated with various concentrations of NPCDs (0-30 μL/mL). After 24 h of incubation, the cells were washed with PBS and stained with Rhodamine-123 (1 μg/mL) for 30 min. Epifluorescence images of the cells were obtained via an inverted microscope (Nikon Eclipse TS200, Nikon Corp., Japan) 4.

### Western blot analysis

The B16F10 melanoma cancer cells were cultured in a 3 mL culture dish and then subjected to 24-h NPCDs (0-30 μL/mL) treatment. Furthermore, the cells were washed with PBS, lysed using a radio-immunoprecipitation assay lysis buffer, and centrifuged at 12,000 rpm for 15 min at 4 °C. The obtained supernatant was used for Western blotting after protein quantification. The protein sample (25 μg) was subjected to a 12% SDS-PAGE (sodium dodecyl sulfate-polyacrylamide gel electrophoresis) and then transferred onto a polyvinylidene fluoride (PVDF) membrane, with which 5% skimmed milk was used for membrane blocking. This was incubated with specific antibodies overnight at 4 °C and further visualized using enhanced Western blot chemiluminescence detection reagents (Amersham Biosciences Inc., Piscataway, NJ, USA). Primary and secondary antibodies used in the study are presented in Table S1.

### Detection and quantification of autophagic vacuole formation

Briefly, B16F10 cells (1×10^4^ cells/well) were seeded in 24-well plates and allowed to adhere overnight. After treatment with different concentrations of NPCDs (0-30 μL/mL) for 24 h, autophagic vacuoles were detected with the help of acridine orange (AO) staining (100 μg/mL). Fluorescence images were captured under the inverted microscope at 40× magnification. Moreover, lysosomal activity was evaluated with the LysoTracker VR Red autophagy detection kit (Enzo Life Sciences, Plymouth Meeting, PA, USA) according to the manufacturer's instructions. DAPI (4′,6-diamidino-2-phenylindole) was used as a counterstain for the nuclei. Fluorescence images were captured under the inverted microscope and the mean fluorescence intensity was quantified using the ImageJ software.

### siRNA transfection studies

The human Atg5 siRNA (5′-ACGCUAAAAGGCUUACAGUAUCAGA-3′) was synthesized by integrated DNA technology (Iowa, USA). Non-specific control siRNA duplexes (Integrated DNA Technologies, Iowa, USA) were also used in parallel experiment. For the experiment, before transfection, 1 × 10^5^ B16F10 melanoma cells were seeded in 6-well plates and grown in 3 mL of RPMI-1640 supplemented with 10% fetal bovine serum and antibiotics. The siRNA or control duplexes were transfected into cells with trans-IT-TKO transfection reagent and incubated for 48 h. Next, cells were harvested for RNA isolation and evaluated for the expression of *atg5*.

### Isolation of RNA and quantitative real-time PCR (qRT-PCR)

Total RNA was isolated from B16F10 cells using an RNA-spinTM extraction kit (Intron Biotechnology, Korea) as per the manufacturer's instructions and quantified by a Qubit22s 2.0 fluorometer RNA assay kit (Life technologies, USA). cDNA synthesis was carried out using a Maxime RT Premix cDNA synthesis kit (Intron Biotechnology, Korea) according to the manufacturer's instructions. Finally, qRT-PCR was performed using an Agilent technology qPCR system (CA USA). Details of the primers used in the study are given in Table S2.

### Cellular bioimaging of B16F10 cells

We report highly fluorescent NPCDs suitable for biological labeling and imaging applications. For living cell staining experiments, B16F10 melanoma cells cultured initially were seeded into 24-well plates and incubated at 37 °C in a 5% CO_2_ environment for 12-24 h. Subsequently, NPCDs (0-10 μL/mL) were injected into each treatment well (non-treated wells served as the control), followed by incubation for 30 min. The cells were washed twice or thrice with PBS after incubation. Epifluorescence images of the cells with/without NPCDs were obtained under an inverted microscope (ex: green 500-570 nm; red 610-750 nm, and blue 450-500 nm; Nikon Eclipse TS200, Nikon Corp., Tokyo, Japan). Each experiment was repeated three times (independently), and at least three different fields were observed for each culture 38.

### Statistical analysis

All experiments were performed in triplicate. The results are expressed as the mean ± standard deviation of three independent experiments. Statistical significance was calculated using Student's *t*-test (*p < 0.05, **p <0.01, *** *p* < 0.001).

## Results

### Characterization

**Figure 1:**
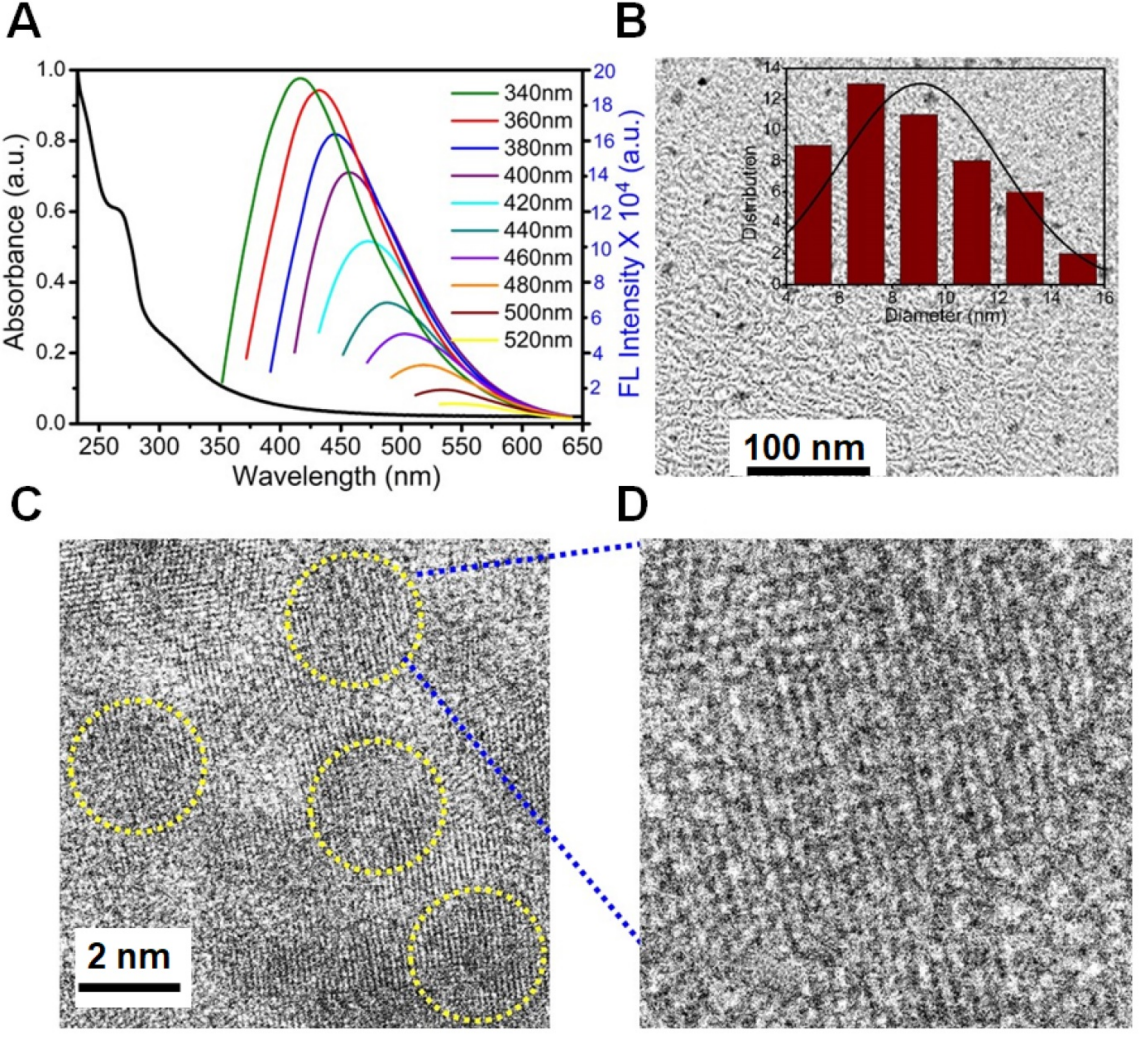
Optical and morphological characterization of nitrogen-phosphorous-doped carbon dots (NPCDs). (A) UV-visible and fluorescence emission spectra, (B) a low-resolution transmission electron microscopy (TEM) image with size histogram in inset image, (C and D) high-resolution TEM images.

**Figure 2:**
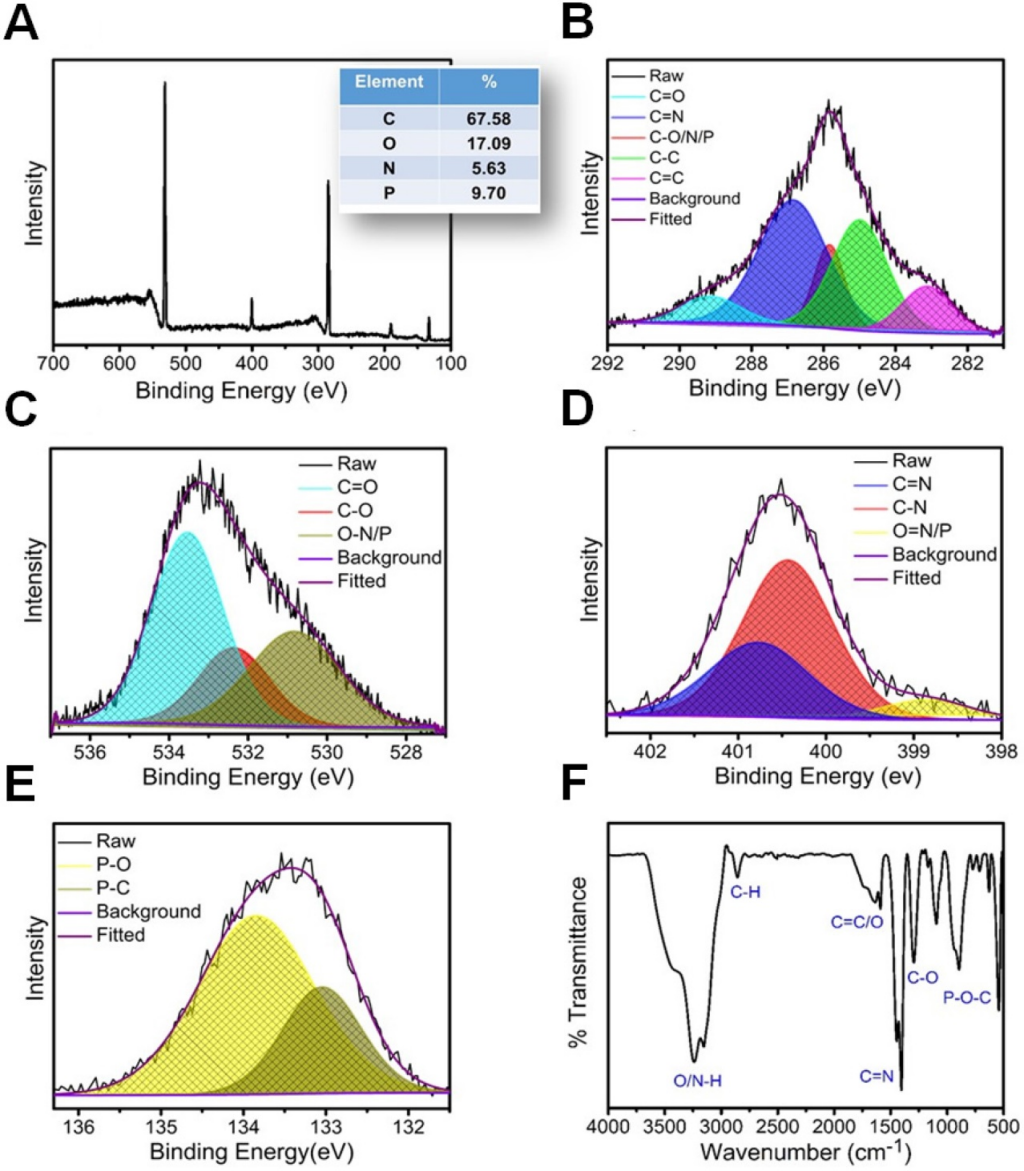
Structural characterization of nitrogen-phosphorous-doped carbon dots (NPCDs). (A) Full survey XPS scan with elemental composition; the corresponding deconvoluted spectra of (B) C_1s_, (C) O_1s_, (D) N_1s_, and (E) P_2p_ short scans; and (F) FTIR spectra of NPCDs.

A merge graph of absorption and fluorescence spectra of NPCDs is shown in Figure 1A. The absorption spectra exhibited two bands at ~275 nm (π-π*) and ~320 nm (n-π*) due to the emergence of C=C and C=N/O/P groups, respectively 20, 39, 40. The fluorescence spectra of NPCDs showed excitation wavelength-dependent spectra (340-520 nm) with emissions in the blue-green region (Max. = ~ 425 nm) 41. The core structure and morphology of NPCDs suggested the spherical shape of particle with an average diameter of 6-8 nm, as observed from the TEM analysis (Figure 1B and inset). The HRTEM image of the homogeneously distributed NPCDs are shown by the yellow highlighted circles (Figure 1C) and displayed the graphitic pattern and interplanar spacing of 0.23 nm (Figure 1D). Further, the surface functional groups and doping of each atom were characterized by the XPS and FTIR spectroscopy, respectively. The full survey scan of NPCDs showed the presence of different atoms as a peak at ~285.02 eV for carbon (67.58 %), at ~531.8 eV for oxygen (17.09 %), at ~399.9 eV for nitrogen (5.63 %), and at ~190.1 and ~133.5 eV for phosphorus (9.7 %) from C_1s_, O_1s_, N_1s_, P_2s_ and P_2p_, respectively (Figure 2A). Figure 2B displays C_1s_ short-scan spectra that were exhibiting five different bindings as a peak at ~283.07 eV, which corresponds to the binding C=C, ~284.9 eV for C-C, ~285.8 eV for C-O/N/P, ~286.8 eV for C=N, and ~289.2 eV for C=O binding 42. The different types of oxygen bindings were observed in O_1s_ short scan spectra such as O-N/P at 530.7 eV, C-O at 532.6 eV, and C=O at 533.5eV (Figure 2C). N_1s_ spectra were showing three different bindings of O=N-C at 398.8 eV, C-N at 400.0 eV, and C=N 400.8 eV confirming the doping of N element (Figure 2D). P_2p_ short scan spectra showing two different types of binding as P-O and P-C bonds at ~133.8 eV and ~133 eV (Figure 2E), confirmed the doping of P atom 39. Moreover, the FTIR spectrum showing the functional groups O/N-H, C-H, C=O/C=C, C=N, C-O, and P-O-C vibrations occurred at peak position ~3360/3155 cm^-1^, ~2854 cm^-1^, ~1735-1581 cm^-1^, ~1440.5 cm^-1^, ~1292 cm^-1^, ~1092-820 cm^-1^ wavenumber, respectively (Figure 2F) 28, 29.

### Anticancer effects of the NPCDs

#### NPCDs promote cytotoxicity in B16F10 cells

**Figure 3:**
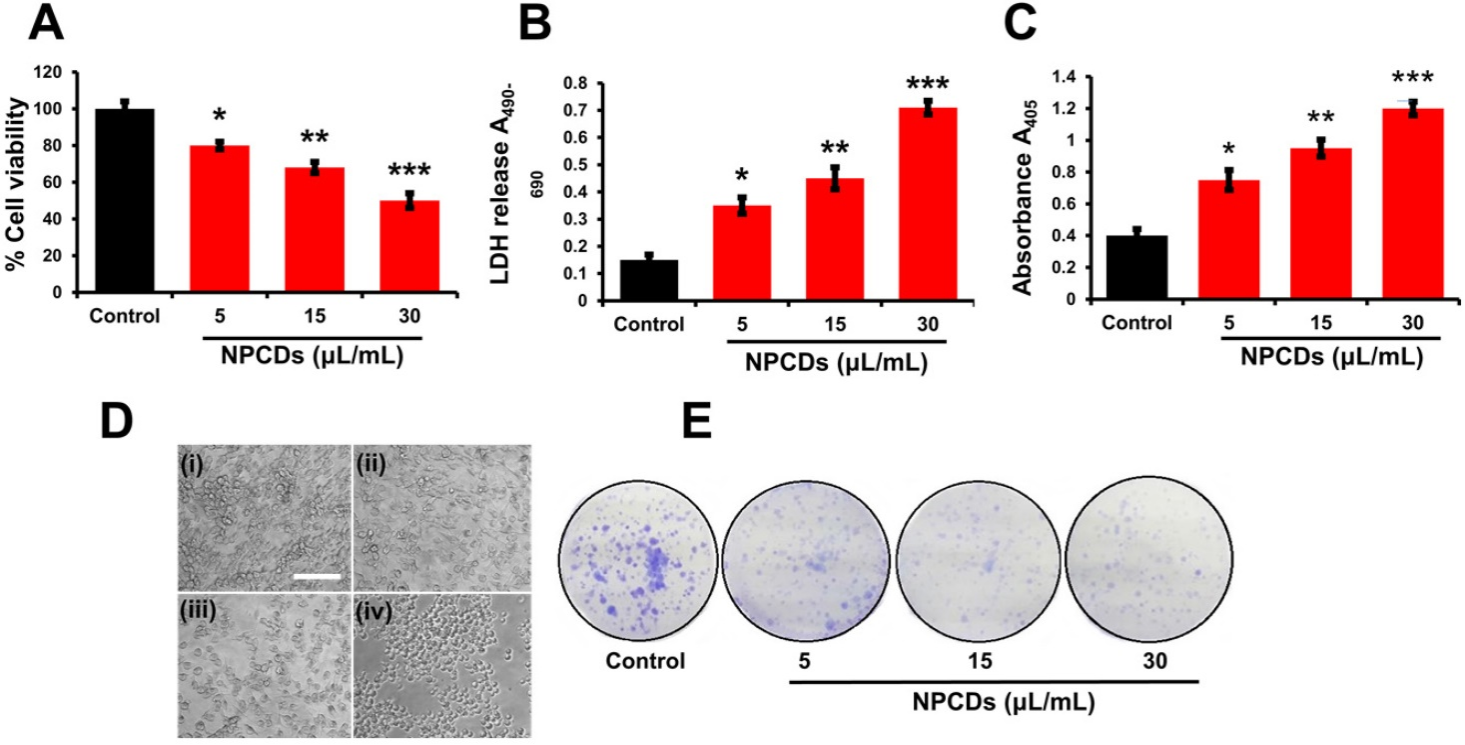
Cytotoxic potential of nitrogen-/phosphorous-doped carbon dots (NPCDs) against B16F10 melanoma cells. (A and B) MTT and LDH assay, respectively of the NPCDs (0-30 μL/mL)-treated cells after 24 h of incubation. (C) Apoptosis assay after treatment with NPCDs measured at A_405_ (D) Morphological changes in B16F10 cells after treatment with NPCDs (0-30 μL/mL). Images were captured at 20× magnification [scale bar =0.1 mm]. (E) Clonogenic assay where B16F10 cells were cultured in the presence and absence of NPCDs over 7 days, and then subjected to crystal violet staining. Each value in the bar graph represents the mean ±SD of three independent experiments. Values with different superscripts differ significantly from each other (*P* < 0.05).

**Figure 4:**
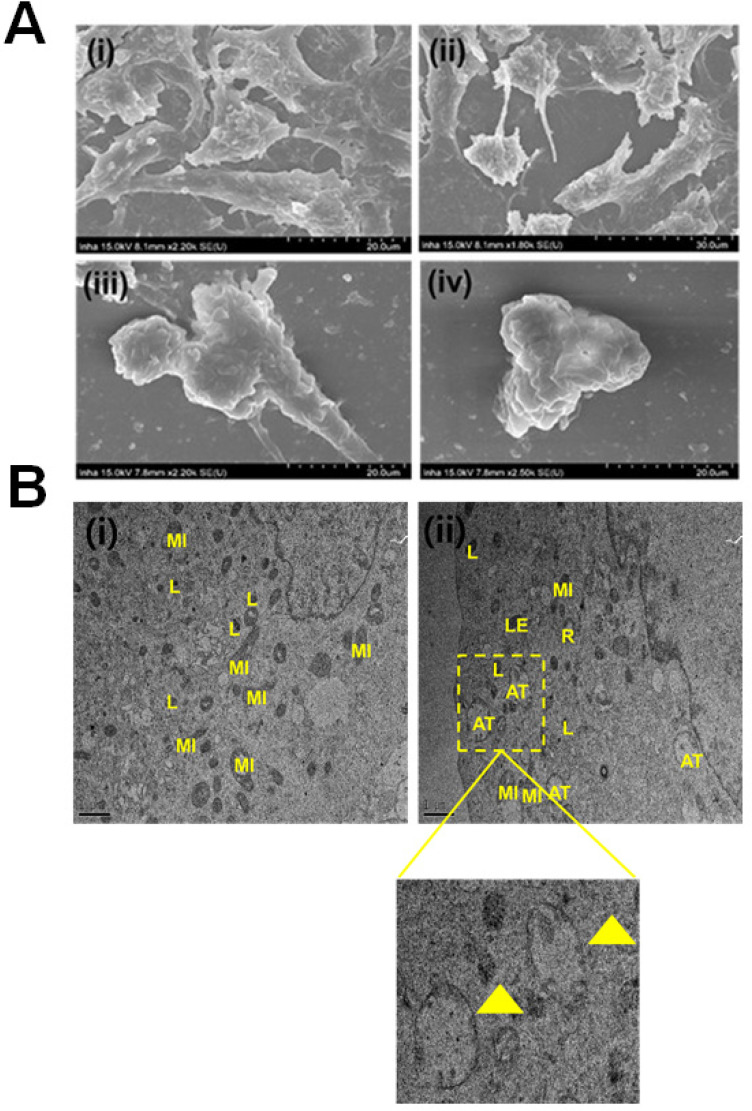
Evaluation of the morphological and internal cellular damage to B16F10 melanoma cancer cells after treatment with nitrogen-/phosphorous-doped carbon dots (NPCDs) via scanning electron microscopy (SEM) and bio- transmission electron microscopy (bio-TEM) analysis. (A) SEM images of the (i) control and (ii) 5 μL/mL, (iii) 15 μL/mL and (iv) 30 μL/mL NPCD-treated B16F10 cells; and (B) Bio-TEM images of the (i) control and (ii) 30 μL/mL NPCD-treated B16F10 cells. L, lysosome; LE, late lysosome; V, vacuole; MI, mitochondrion; and R: ribosome (Scale: 1 μm).

Primarily, we performed MTT assay with various cancer cell lines in the presence of NPCDs (0-120 µL/mL) for 24 h, and the values obtained from MTT assay results were utilized to construct the heatmap as depicted in Figure S1, and based on the best results obtained, we further selected melanoma cancer cell (B16F10) in subsequent assays for rest of the study. The MTT assay revealed that 30.01 μL/mL of NPCDs served as the fifty percent inhibitory concentration (IC_50_) against B16F10 cells (Figure S2). The MTT assay (Figure 3A) and LDH (Figure 3B) assays proved the dose-dependent anticancer potential of NPCDs against B16F10 cells (Figure 3). Furthermore, NPCDs did not show any serious cytotoxic effect on both the tested normal cells (human keratinocytes HACAT and BEAS-2B lung epithelial cells) (Figure S3). Moreover, we determined whether NPCD-induced DNA fragmentation, which is characteristic of apoptosis, or the lack thereof by performing the ApoStrand ELISA assay. The results of this assay confirmed distortion in the chromatin structure, which is associated with apoptosis (Figure 3C). The dose-dependent effect of NPCDs on cell morphology is depicted in Figure 3D, which showed distortion and killing of the NPCDs-treated cells, and rupturing of cell membrane. The cytotoxicity was further validated via clonogenic assay, which confirmed reduced colony formation after treatment with NPCDs (Figure 3E). Additional validation was obtained through SEM analysis, which revealed the distorted morphology of the B16F10 cell at an NPCD concentration of 30 µL/mL compared with that of the control group with intact cell morphology (Figure 4A). Moreover, the results of a bio-TEM analysis showed broken vacuoles and decreased numbers of mitochondria, lysosomes, late lysosomes, vacuoles, ribosomes as well as autophagosome formation (Figure 4B). These results collectively suggest the cytotoxic activity of the NPCDs against the B16F10 cells.

#### NPCDs mediate apoptotic morphology in B16F10 cells

**Figure 5:**
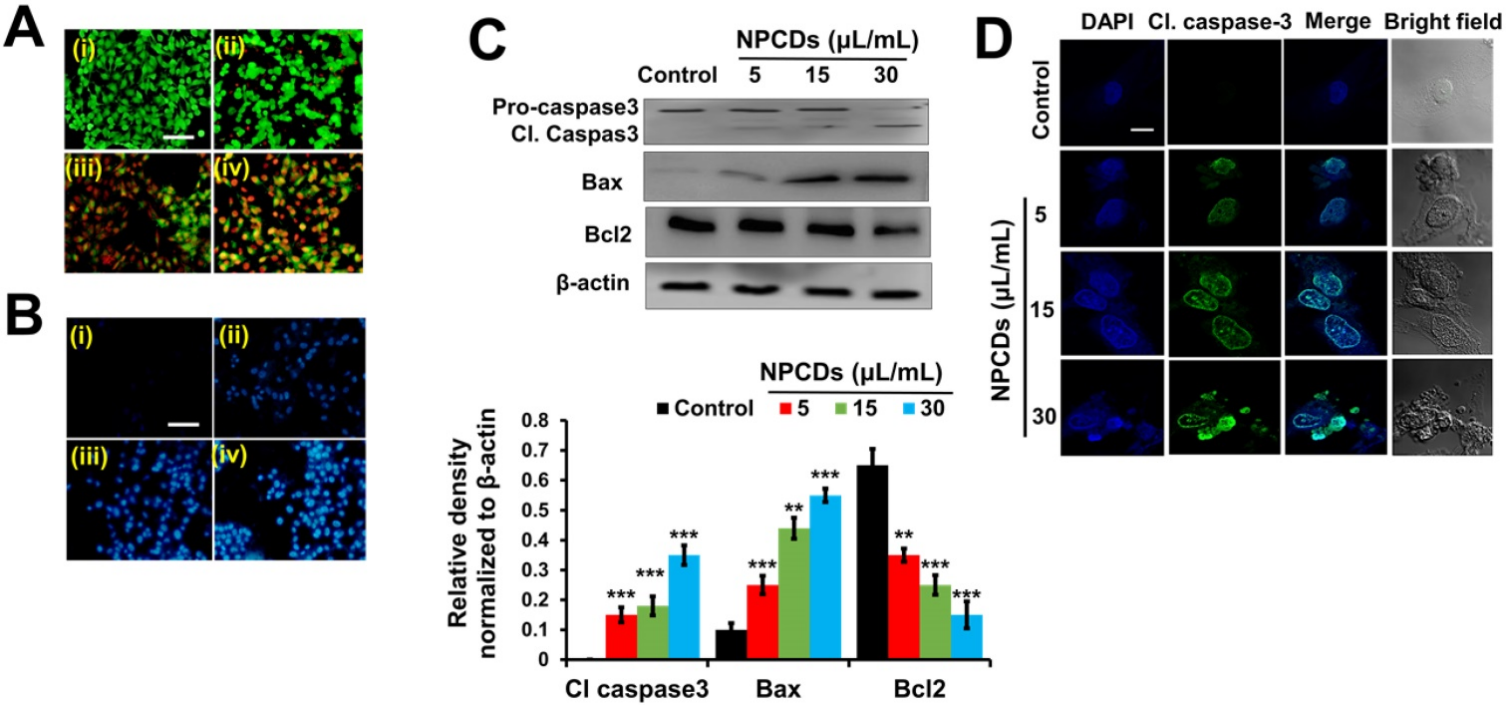
Nitrogen-/phosphorous-doped carbon dots (NPCDs; 0-30 μL/mL)-induced apoptotic morphology in B16F10 melanoma cancer cells. Apoptotic morphology was evaluated by A) live-dead cells and B) Hoechst-PI staining. (C) Western blot analysis of various apoptotic markers (caspase-3, Bcl2, and Bax) and Image J analysis. (D) Immunocytochemistry of prominent apoptosis marker cleaved caspase-3 after treatment with NPCDs evaluated via confocal microscopy. The data are represented as the mean ± standard deviation (SD) of three independent experiments: ***P < 0.001, **P < 0.01, * *P* < 0.05 vs. the control. Fluorescence images were captured at 20× magnification (scale bar = 0.1 mm).

The ability of the NPCDs to induce apoptosis and the stages thereof was determined with the help of dual AO/ethidium bromide staining, with which the normal cells and the apoptotic cells appear green and orange (or red), respectively. The results revealed that the NPCDs caused a dose-dependent increase in the number of apoptotic cells. For example, the apoptotic index of the cells treated with 30 μL/mL NPCDs was significantly (*p* < 0.05), i.e. 7.5-fold higher than that of the control (Fig 5A). We validated the results via Hoechst- PI staining, which indicates the enhancement of dead and apoptotic cells, and the elevated red fluorescence depicted DNA damage and chromatin condensation in a dose-dependent manner. The number of apoptotic cells among those treated with 30 μL/mL NPCDs was significantly higher (*p* < 0.05; 5.6-fold) than those in the control (Figure 5B). Furthermore, the NPCD-induced apoptosis was confirmed by immunoblotting. We detected the 6-fold (*p* < 0.001), 4.5-fold (*p* < 0.001), and 7.5-fold (*p* < 0.001) enhancement of cleaved caspase-3, cleaved caspase-9, and Bax (B-cell lymphoma 2-associated X) protein level expression, respectively (Figure 5C), and we also recorded a reduction in Bcl2 (B-cell lymphoma 2) protein expression level in a dose-dependent manner (Figure 5C and 5D). Furthermore, to validate our results, we detected the cleaved caspase-3 levels in the cells by using an immunofluorescence assay and observed a dose-dependent increase (Figure 5E).

#### Oxidative burst and mitochondrial membrane potential

**Figure 6:**
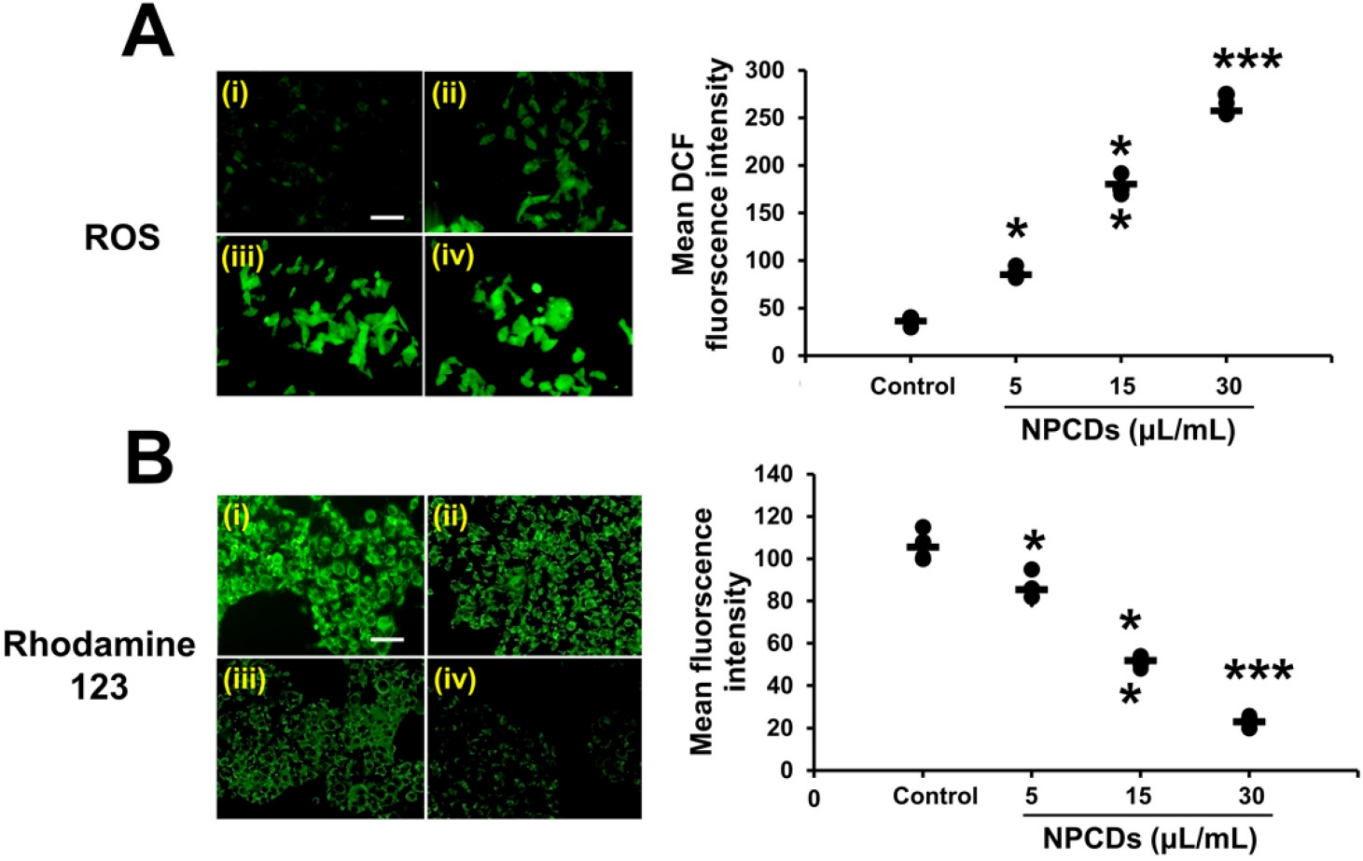
Nitrogen-/phosphorous-doped carbon dots (NPCDs; 0-30 μL/mL)-induced cytosolic and mitochondrial ROS production in B16F10 melanoma cancer cells. (A) cytosolic ROS production determined by H_2_DCFDA counterstaining with DAPI and (B) Rhodamine 123 staining detected by fluorescence microscopy. Fluorescence intensities of (C) cytosolic and (D) mitochondrial ROS showing results corresponding to (i) the control and (ii) 5, (iii) 15, and (iv) and 30 μL/mL of NPCDs. ImageJ software was used to plot their respective intensities. Fluorescence images were captured at 20× magnification (scale bar = 0.1 mm). The data are represented as the mean ± standard deviation (SD) of three independent experiments: ***P < 0.001, **P < 0.01, * *P* < 0.05 vs. the control.

Our results obtained via H_2_DCFDA staining of B16F10 cells indicate that the oxidative stress was enhanced, as manifested by the dose-dependent increase in green fluorescence after treatment with NPCDs (Figure 6A), although the oxidative stress yielded a decrease in the mitochondrial membrane potential (Δψm). Similarly, the Rhodamine-123 fluorescence decreased in a dose-dependent manner in the B16F10 cells (Figure 6B).

#### NPCDs provoke cell cycle arrest in B16F10 cells

**Figure 7:**
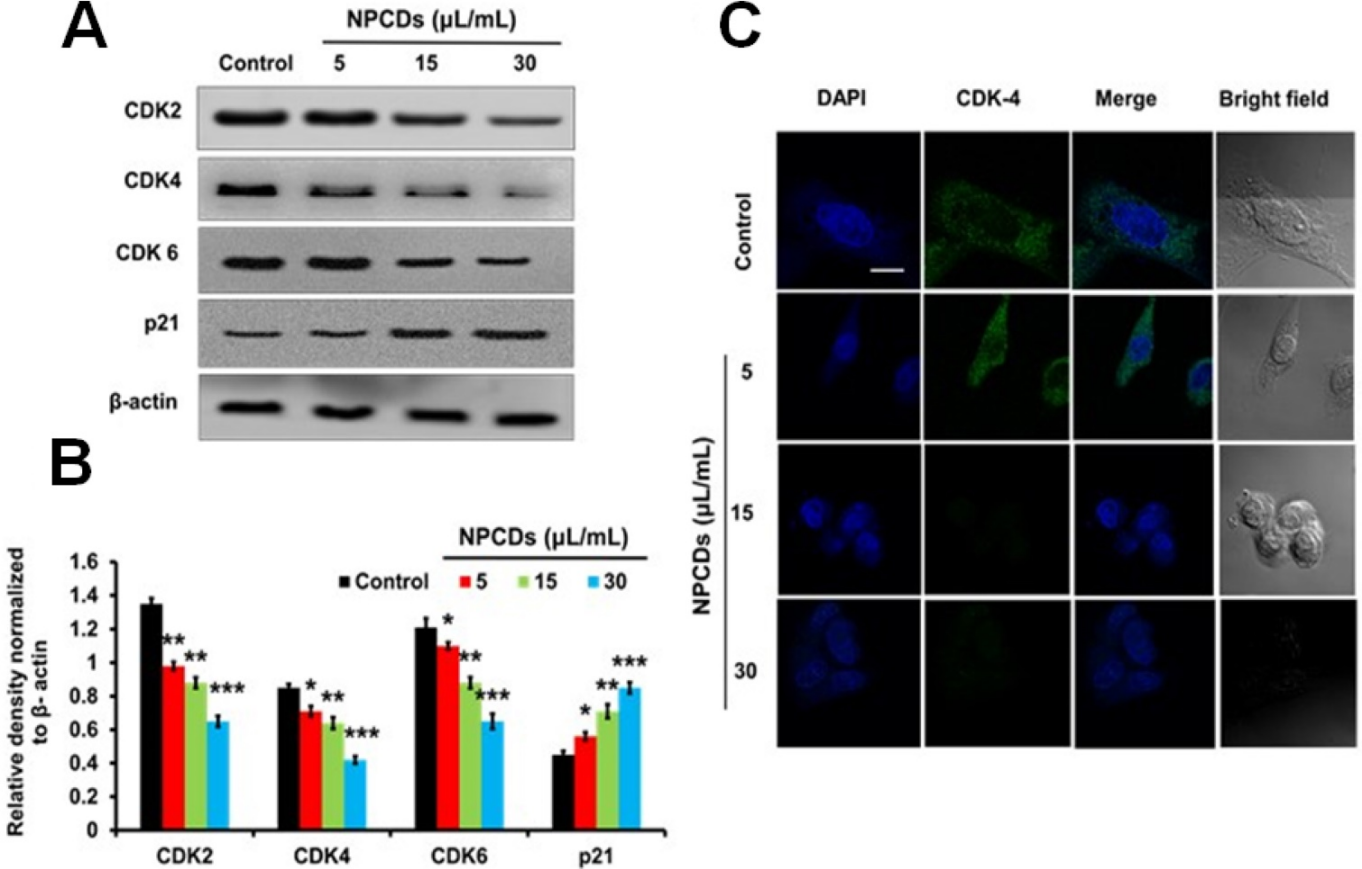
Effect of nitrogen-/phosphorous-doped carbon dots on cell cycle arrest markers. (A) Western blot analysis of various cell cycle arrest markers (CDK-2, -4, and -6 and p21) and (B) ImageJ analysis of B16F10 melanoma cancer cells after treatment with nitrogen-/phosphorous-doped carbon dots (NPCDs; 0-30 μL/mL). (C) Immunocytochemistry of cell cycle arrest marker CDK-4 after treatment with NPCDs evaluated via confocal microscopy. The data are represented as the mean ± standard deviation (SD) of three independent experiments: ***P < 0.001, **P < 0.01, * *P* < 0.05 vs. the control.

The role of NPCDs in cell cycle arrest was evaluated by examining the expression levels of CDK-2, -4, and -6 and p21. Figure 7A reveals a significant (p < 0.05) dose-dependent reduction in the expression levels of CDK-2, -4, and -6 and a marked increase in p21 expression in the B16F10 cells treated with NPCDs (Figure 7B). All of the results collectively suggest that the NPCDs induce cell senescence and cell cycle arrest in B16F10 cells. Furthermore, the obtained results of immunoblotting were further validated by using an immunofluorescence assay. We detected a dose-dependent reduction in fluorescence of CDK-4 counterstained with DAPI (Figure 7C).

#### NPCDs induce autophagy in B16F10 cells

**Figure 8:**
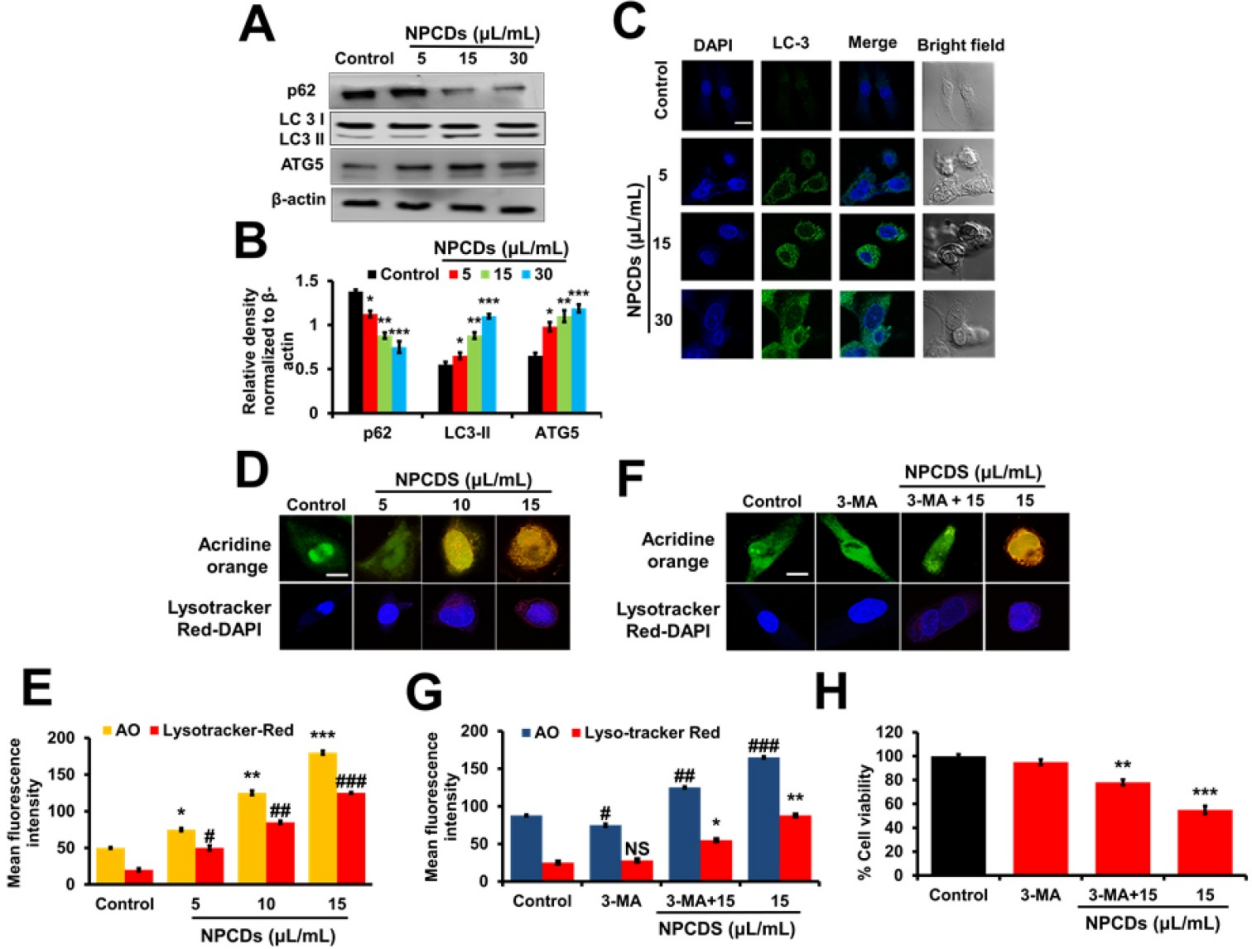
Effect of nitrogen-/phosphorous-doped carbon dots on autophagy markers. (A) Western blot analysis of various autophagy markers (LC3, ATG-5, and p62) and (B) ImageJ analysis of B16F10 melanoma cancer cells after treatment with nitrogen-/phosphorous-doped carbon dots (NPCDs; 0-30 μL/mL). (C) Immunocytochemistry of prominent autophagy marker LC3 after treatment with NPCDs evaluated via confocal microscopy. (D) Autophagic vacuole formation after treatment with NPCDs detected via confocal microscopy. (E) ImageJ analysis of autophagic vacuole formation. (F and G) Validation of autophagic vacuole formation evaluated in the presence of 3-methyladenine ((3-MA) preincubated with 2.5 mM for 1 h and then incubated with NPCDs for 24h). The data are represented as the mean ± standard deviation (SD) of three independent experiments: ***P < 0.001, **P < 0.01, * *P* < 0.05 vs. the control; ^###^*P* < 0.001, ^##^*P* < 0.01, ^#^ *P* < 0.05 vs. the control.

The role of autophagy in cell survival and death has been reported in several studies. Therefore, to further investigate the role of the NPCDs in this process, we evaluated the level of crucial autophagic markers such as LC3 (microtubule-associated protein 1A/1B-light chain 3), which revealed the elevated dose-dependent expression level of the LC3-phosphatidylethanolamine (LC3-II) marker (Figure 8A). The results revealed that, in addition to LC3-II and ATG-5 (autophagy-related-5) also being upregulated in a dose-dependent manner (Figure 8A), NPCD treatment also resulted in sequestosome-1 (p62/SQSTM1) degradation. These results indicate that NPCDs lead to autophagic cell death of B16F10 melanoma cells (Figure 8B). Furthermore, the obtained immunoblotting data was confirmed by additional experiments such as immunofluorescence measurements. We detected the dose-dependent increase in LC3-II fluorescence counterstained with DAPI, which is a hallmark of autophagy, after treatment with the NPCDs (Figure 8C). Moreover, we used AO and LysoTracker Red to detect the autophagy levels (Figure 8D and 8E) and validated the results with 3-MA (methyladenine) as an ATG-5 inhibitor (Figure 8F and 8G). The obtained results indicate that the NPCDs induce autophagy in a dose-dependent manner, as validated by the autophagy inhibitor.

#### Autophagy attenuation

To determine the role of autophagy after induction with NPCDs, we did gene silencing of *atg5* by using siRNA and evaluated normalized gene expression (*atg5, lc-3, p62,* and* caspase-3*) by real-time PCR analysis. Knockdown was executed to down-regulate *atg5*, an important molecule for the initial formation of autophagosome and results were determined by real-time PCR (Figure S4). Our obtained data denoted that *atg5* was significantly down-regulated by siRNA at 48 h detected by RT-PCR. This down-regulation of *atg5* also suppressed autophagy which was evident by reduced *lc-3* gene levels in B16F10 cells. When *atg5* silenced cells were treated in the presence of NPCDs, *lc-3* and *p62* levels were also increased compared to the *atg5* silenced cells. Moreover, we also observed no change of *caspase3* in *atg5* silenced group compared to control, however, similar levels of *caspase3* gene were observed under NPCDs as well as combination of *atg5* siRNA and NPCDs-treated group compared to control group, which indicated that cell death occurred by apoptotic pathway in B16F10 cells even after knockdown of *atg5*.

### Application of NPCDs as nanocarriers in cellular labeling/imaging

**Figure 9:**
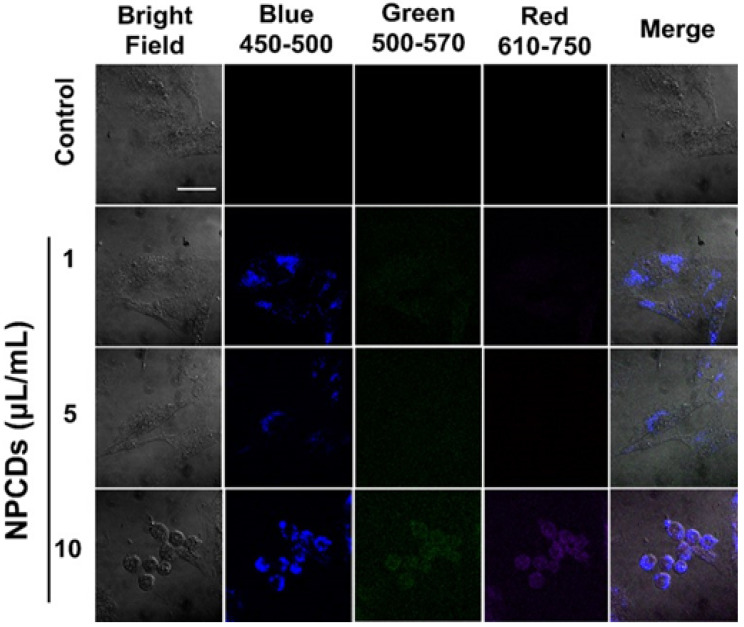
Application of nitrogen-/phosphorous-doped carbon dots (NPCDs) in B16F10 melanoma cell bioimaging. Treatment of the B16F10 melanoma cancer cells gave rise to dose-dependent green, red, and blue fluorescence after 30 min of incubation in the dark. Cells emitting blue, green, and red fluorescence at, ex: 450-500 nm, ex: 500-570 nm and ex: 610-750 nm.

We used the highly fluorescent NPCDs for cellular imaging. As depicted in Figure 9, treatment of the B16F10 melanoma cells with NPCDs at different concentrations (1, 5, and 10 µL/mL) was found to be very effective in cell bioimaging, as confirmed by the cells emitting green, red, and blue fluorescence at ex: 500-570 nm, ex: 610-750 nm, and ex: 450-500 nm, respectively, under an inverted microscope (Figure 9); treatment with the fluorescent NPCDs made the cells easily visible. At 1 and 5 µL/mL NPCD concentrations, the green, red, and blue fluorescence were found to be slightly enhanced, but it was found to be the highest at 10 µL/mL. We detected the fluorescence from 450 to 750 nm and observed the dose-dependent enhancement of fluorescence.

## Discussion

Skin cancer is the most commonly diagnosed cancer in the US, and most cases are preventable 43, 44. Skin cancer greatly affects the quality of life, and it can be disfiguring or even deadly 43. Medical treatment for skin cancer creates substantial healthcare costs for individuals, families, and the nation. The number of Americans who have had skin cancer at some point in the last three decades is estimated to be higher than the number for all other cancers combined 30-45, and skin cancer incidence rates have continued to increase in recent years 43-46.

Each year in the US, nearly 5 million people are treated for all skin cancers combined, with an annual cost estimated at $8.1 billion 47. Among all skin cancers, melanoma is responsible for the most deaths, with nearly 9,000 each year 48. It is also one of the most common types of cancer among US adolescents and young adults 49. Around $3.3 billion of annual skin cancer treatment costs are attributable to melanoma cancer 47.

A recent addition to the carbon nanomaterial family, quantum CDs, have attracted great interest because of their outstanding water solubility, high sensitivity and selectivity for target analytes, strong disease detection ability, low toxicity, favorable biocompatibility, and excellent photostability 50. Various kinds of quantum CDs have been used as biomedical and biocontrol agents 51. Quantum dots (QDs) are generally semiconductor nanocrystals of physical dimensions smaller than the exciton Bohr radius 52. Because of the quantum-confinement effect, the electronic and optical properties of QDs exhibit characteristic dependencies on nanoparticle size and composition, especially the infamous display of beautiful fluorescence colors for QDs of varying semiconductor nanoparticle sizes. The high optical performance of QDs has captured the imagination of researchers in biomedical fields, with the exploration of a wide variety of potential bio-applications already producing exciting results. However, there have not yet been any reports on the anticancer activity of doped QDs. Therefore, in this study, the NPCDs were synthesized using PEG as a C source, imidazole as an N source, and phosphoric acid as a P source. They were then characterized by FTIR, XPS, UV-Vis, HRTEM, and fluorescence analyses.

In our study, we employed the NPCDs to evaluate their anticancer potential on B16F10 melanoma cells. Initially, we explored their anticancer as well as apoptotic activity and found that the NPCDs exert apoptotic activity on B16F10 cells. Moreover, apoptosis is a highly energy-dependent process governed by activation and or inactivation of myriad arrays of proteins 3. It is crucial to understand the complex process of apoptosis, which is comprised of DNA degradation, chromatin condensation, and shrinkage of cell, as well as blebbing 3. We found that the NPCDs caused apoptotic cell and apoptotic nuclei formation, which is in accordance with a previous report on the apoptotic potential of carboxylic acid-coated CDs against human keratinocytes 53.

Under stressful conditions, cells undergo much cellular orchestration to organize many cellular signaling cascades, including activation of oxidative stress 4, 37 which is the main protagonist of most apoptotic cell death. Activated ROS not only function to activate further cellular events but also create an imbalance among various organelles such as mitochondria 37. The disturbed homeostasis of the mitochondria causes dysregulation of Bax and Bcl2 protein levels 54. Moreover, oxidative imbalance causes weakening of the mitochondrial membrane, leading to the release of cytochrome C from the mitochondria 50, this phenomenon is very well known for insinuating the activation of proteases such caspases, thereby leading to apoptotic cell death 55. In this study, we found that the NPCDs caused B16F10 cells to undergo oxidative stress and then mitochondrial-mediated caspase-dependent apoptosis. Similarly, Nguyen et al. 56 reported that exposure to cadmium telluride QDs led to oxidative stress and apoptosis in HepG2 cells.

The cell cycle is composed of four phases by activation or inactivation of cyclin-dependent kinases depending upon the status of the cell and the surrounding environment 51. The initial phase involves cyclin D1, CDK-4, and CDK-6 that help to build the G1 phase, which is known to be crucial for the formation of the cell cycle. The cell then enters the synthesis phase where cyclin E and CDK-2 are needed for DNA synthesis. In the subsequent G2 to M transition phase, the Cdc2 (cell division control protein kinase 2)-cyclin B complex is required. Many commercially available drugs hamper the cell cycle, leading to cell death. Similarly, the NPCDs were found to arrest the cell cycle by downregulating the CDK complex and upregulating p21 protein levels. Relatedly, graphene QDs were also reported by Sui et al. 51 to be responsible for causing cell cycle arrest and cell death.

**Figure 10:**
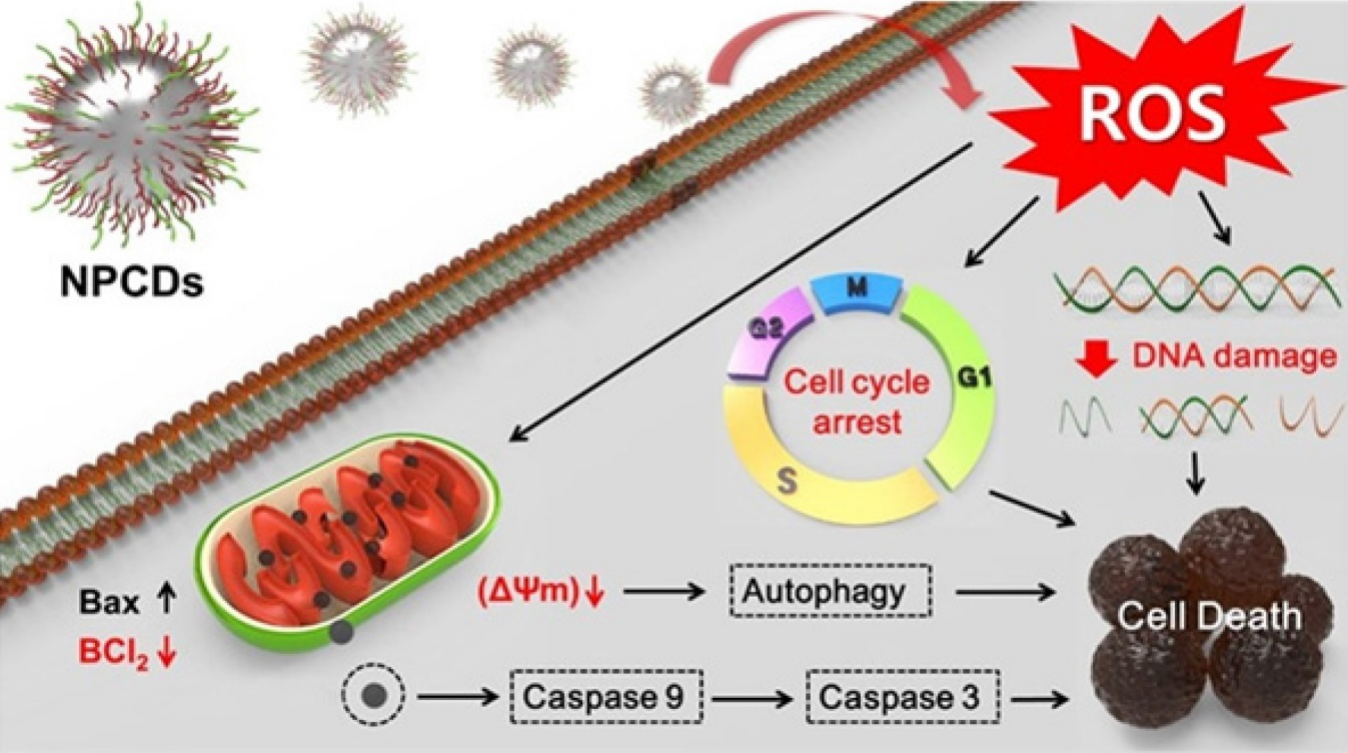
Anticancer mechanism of nitrogen-/phosphorous-doped carbon dots (NPCDs) against B16F10 melanoma cells.

Along with the cell cycle and apoptosis, autophagy is also responsible for causing cell death or survival depending upon the cell fate 57. Autophagy is a lysosomal catabolic process utilized by cells to eradicate damaged and non-functional proteins or metabolites 6. In the past few years, research has revealed that autophagy has a greater variety of pathophysiological functions than was previously known 6. Mitochondrial membrane weakening also triggers the autophagic process, subsequently leading to autophagic cell death 58. Similarly, we explored the role of NPCDs in autophagic cell death and the overall mechanism of their anticancer effects is presented in Figure 10.

Based on the non-toxic and remarkable fluorescence performance of the NPCDs in our study, *in vitro* cellular imaging was conducted on B16F10 melanoma cells. The distinctive advantages of CDs are their multicolor emission profile, small size, low cytotoxicity, prominent biocompatibility, and excellent photostability, making them ideal candidates for fluorescence imaging 59. As expected, strong green and red fluorescence were obtained at the wavelengths of green (500-570 nm), red (610-750 nm), and blue (450-500 nm) from the NPCD-labeled B16F10 melanoma cancer cells. This indicates that the NPCDs were effectively taken up by the B16F10 melanoma cancer cells and could be used as fluorescent probes for cellular imaging that addresses many issues in different fields of research, such as alternatives for chemical substances commonly used to stain (dye) drug delivery and cell labeling. Similarly, Vasimalai et al. 60 also explored the use of CDs as high-performance, non-toxic fluorescence agents for optical *in vitro* bioimaging of cancer cells such as human glioblastoma cells (the LN-229 cancer cell line). In general, staining cancer cells is important because without it, most cancer cells are extremely difficult to see. Staining allows them to be seen so that observations as to their morphology (i.e. individual cell shape) and arrangement (i.e. how the cells remain physically attached to one another after cell division) can be made. Based on these results, we can assume that the NPCDs could be effectively used for cancer cell imaging 12, 61.

## Conclusions

To summarize, we herein report the synthesis and characterization of multifunctional and biocompatible NPCDs by means of a green thermal treatment method. We demonstrated the significant anticancer potential of NPCDs on a melanoma cell line and explored the pathways that allow the NPCD mediation of cell death. More precisely, we found that the NPCDs regulate the cell death pathway by inducing apoptosis, cell cycle arrest, and autophagy. Application of NPCDs for cellular bioimaging of B16F10 melanoma cells further validated their usefulness in the diagnosis of cancer-associated diseases. Therefore, our green-synthesized NPCDs could be useful in the detection and treatment of skin cancer, although *in vivo* studies are needed to further validate their practical application.

## Supplementary Material

Supplementary figures and tables.

## References

[1] Apalla Z, Lallas A, Sotiriou E, Lazaridou E, Ioannides D (2017). Epidemiological trends in skin cancer. Dermatol Pract Concept.

[2] Bisht G, Rayamajhi S (2016). ZnO nanoparticles: a promising anticancer agent. Nanobiomedicine.

[3] Elmore S (2007). Apoptosis: a review of programmed cell death. Toxicol Pathol.

[4] Khan I, Kang SC (2017). Apoptotic activity of *Lactobacillus plantarum* DGK-17-fermented soybean seed extract in human colon cancer cells via ROS-JNK signaling pathway. J Food Sci.

[5] Khan I, Bahuguna A, Krishnan M, Shukla S, Lee H, Min S-H (2019). The effect of biogenic manufactured silver nanoparticles on human endothelial cells and zebrafish model. Sci. Total Environ.

[6] Glick D, Barth S, Macleod KF (2010). Autophagy: cellular and molecular mechanisms. J Pathol.

[7] Jog NR, Caricchio R (2014). The role of necrotic cell death in the pathogenesis of immune mediated nephropathies. Clin Immunol.

[8] Tran S, DeGiovanni P-J, Piel B, Rai P (2017). Cancer nanomedicine: a review of recent success in drug delivery. Clin Trans Med.

[9] Zink D, Fischer AH, Nickerson JA (2004). Nuclear structure in cancer cells. Nat Rev Cancer.

[10] Hu Q, Gao M, Feng G, Liu B (2014). Mitochondria-targeted cancer therapy using a light-up probe with aggregation-induced-emission characteristics. Angew Chem Int Ed Engl.

[11] Chao X-J, Wang K-N, Sun L-L, Cao Q, Ke Z-F, Cao D-X (2018). Cationic organochalcogen with monomer/excimer emissions for dual-color live cell imaging and cell damage diagnosis. ACS Appl Mater Interfaces.

[12] Zhao X, Zhang J, Shi L, Xian M, Dong C, Shuang S (2017). Folic acid-conjugated carbon dots as green fluorescent probes based on cellular targeting imaging for recognizing cancer cells. RSC Adv.

[13] Sun Y-P, Zhou B, Lin Y, Wang W, Fernando KS, Pathak P (2006). Quantum-sized carbon dots for bright and colorful photoluminescence. J Am Chem Soc.

[14] LeCroy GE, Sonkar SK, Yang F, Veca LM, Wang P, Tackett KN (2014). Toward structurally defined carbon dots as ultracompact fluorescent probes. ACS Nano.

[15] Park Y, Yoo J, Lim B, Kwon W, Rhee S-W (2016). Improving the functionality of carbon nanodots: doping and surface functionalization. J Mater Chem A.

[16] Yao B, Huang H, Liu Y, Kang Z (2019). Carbon dots: a small conundrum. Trends Chem.

[17] Atabaev TS (2018). Doped carbon dots for sensing and bioimaging applications: A minireview. Nanomaterials.

[18] Anand SR, Bhati A, Saini D (2019). Antibacterial nitrogen-doped carbon dots as a reversible “fluorescent nanoswitch” and fluorescent ink. ACS Omega.

[19] Wang Z-X, Yu X-H, Li F, Kong F-Y, Lv W-X, Fan D-H (2017). Preparation of boron-doped carbon dots for fluorometric determination of Pb (II), Cu (II) and pyrophosphate ions. Microchim Acta.

[20] Bhati A, Anand SR, Saini D, Sonkar SK (2019). Sunlight-induced photoreduction of Cr (VI) to Cr (III) in wastewater by nitrogen-phosphorus-doped carbon dots. NPJ Clean Water.

[21] Barman MK, Jana B, Bhattacharyya S, Patra A (2014). Photophysical properties of doped carbon dots (N, P, and B) and their influence on electron/hole transfer in carbon dots-nickel (II) phthalocyanine conjugates. J Phys Chem C.

[22] Tian T, He Y, Ge Y, Song G (2017). One-pot synthesis of boron and nitrogen co-doped carbon dots as the fluorescence probe for dopamine based on the redox reaction between Cr (VI) and dopamine. Sensors Actuators B: Chem.

[23] Bhati A, Anand SR (2018). Sunlight-induced photocatalytic degradation of pollutant dye by highly fluorescent red-emitting Mg-N-embedded carbon dots. ACS Sus Chem Eng.

[24] Bhati A, Anand SR, Saini D, Khare P, Dubey P, Sonkar SK (2018). Self-doped nontoxic red-emitting Mg-N-embedded carbon dots for imaging, Cu (II) sensing and fluorescent ink. New J Chem.

[25] Liu M (2020). Optical Properties of Carbon Dots: A Review. Nanoarchitectonics.

[26] Khare P, Bhati A, Anand SR (2018). Brightly fluorescent zinc-doped red-emitting carbon dots for the sunlight-induced photoreduction of Cr (VI) to Cr (III). ACS Omega.

[27] Hu C, Yu C, Li M, Wang X, Dong Q, Wang G (2015). Nitrogen-doped carbon dots decorated on graphene: a novel all-carbon hybrid electrocatalyst for enhanced oxygen reduction reaction. Chem Commun.

[28] Gong X, Zhang Q, Gao Y, Shuang S, Choi MM, Dong C (2016). Phosphorus and nitrogen dual-doped hollow carbon dot as a nanocarrier for doxorubicin delivery and biological imaging. ACS Appl Mater Interfaces.

[29] Ananthanarayanan A, Wang Y, Routh P, Sk MA, Than A, Lin M (2015). Nitrogen and phosphorus co-doped graphene quantum dots: synthesis from adenosine triphosphate, optical properties, and cellular imaging. Nanoscale.

[30] Jelinek R (2017). Materials Science Applications of Carbon-Dots. Carbon Quantum Dots: Springer.

[31] Chan KK, Yap SHK, Yong K-T (2018). Biogreen synthesis of carbon dots for biotechnology and nanomedicine applications. Nano-Micro Lett.

[32] Shi B, Su Y, Zhang L, Huang M, Liu R, Zhao S (2016). Nitrogen and phosphorus co-doped carbon nanodots as a novel fluorescent probe for highly sensitive detection of Fe^3+^ in human serum and living cells. ACS Appl Mater Interfaces.

[33] Nie K, Xu S, Duan X, Shi H, Dong B, Long M (2018). Diketopyrrolopyrrole-doped hybrid FONs as two-photon absorbing and dual-emission fluorescent nanosensors for Hg^2+^. Sens Actuators B Chem.

[34] Sahu S, Behera B, Maiti TK, Mohapatra S (2012). Simple one-step synthesis of highly luminescent carbon dots from orange juice: application as excellent bio-imaging agents. Chem Commun.

[35] Chandra S, Laha D, Pramanik A, Ray Chowdhuri A, Karmakar P, Sahu SK (2016). Synthesis of highly fluorescent nitrogen and phosphorus doped carbon dots for the detection of Fe^3+^ ions in cancer cells. Luminescence.

[36] Khan I, Bahuguna A, Kumar P, Bajpai VK, Kang SC (2018). In vitro and in vivo antitumor potential of carvacrol nanoemulsion against human lung adenocarcinoma A549 cells via mitochondrial mediated apoptosis. Sci Rep.

[37] Khan I, Bahuguna A, Bhardwaj M, Pal Khaket T, Kang SC (2018). Carvacrol nanoemulsion evokes cell cycle arrest, apoptosis induction and autophagy inhibition in doxorubicin resistant-A549 cell line. Artif cells Nanomed Biotechnol.

[38] Martynenko I, Litvin A, Purcell-Milton F, Baranov A, Fedorov A, Gun'Ko Y (2017). Application of semiconductor quantum dots in bioimaging and biosensing. J Mater Chem B.

[39] Liu R, Zhao J, Huang Z, Zhang L, Zou M, Shi B (2017). Nitrogen and phosphorus co-doped graphene quantum dots as a nano-sensor for highly sensitive and selective imaging detection of nitrite in live cell. Sens Actuators B Chem.

[40] Gong X, Liu Y, Yang Z, Shuang S, Zhang Z, Dong C (2017). An “on-off-on” fluorescent nanoprobe for recognition of chromium (VI) and ascorbic acid based on phosphorus/nitrogen dual-doped carbon quantum dot. Anal Chim Acta.

[41] Li H, Shao F-Q, Zou S-Y, Yang Q-J, Huang H, Feng J-J (2016). Microwave-assisted synthesis of N, P-doped carbon dots for fluorescent cell imaging. Microchim Acta.

[42] Li J, Jiao Y, Feng L, Zhong Y, Zuo G, Xie A (2017). Highly N, P-doped carbon dots: rational design, photoluminescence and cellular imaging. Microchim Acta.

[43] Lomas A, Leonardi-Bee J, Bath-Hextall F (2012). A systematic review of worldwide incidence of nonmelanoma skin cancer. Br J Dermatol.

[44] Rogers HW, Weinstock MA, Harris AR, Hinckley MR, Feldman SR, Fleischer AB (2010). Incidence estimate of nonmelanoma skin cancer in the United States, 2006. Arch Dermatol.

[45] Stern RS (2010). Prevalence of a history of skin cancer in 2007: results of an incidence-based model. Arch Dermatol.

[46] Jemal A, Saraiya M, Patel P, Cherala SS, Barnholtz-Sloan J, Kim J (2011). Recent trends in cutaneous melanoma incidence and death rates in the United States, 1992-2006. J Am Acad Dermatol.

[47] Guy Jr GP, Machlin SR, Ekwueme DU, Yabroff KR (2015). Prevalence and costs of skin cancer treatment in the US, 2002- 2006 and 2007- 2011. Am J Preven Med.

[48] Group UCSW (2013). United States cancer statistics: 1999-2010 incidence and mortality web-based report. Atlanta: US Department of Health and Human Services, Centers for Disease Control and Prevention and National Cancer Institute.

[49] Weir HK, Marrett LD, Cokkinides V, Barnholtz-Sloan J, Patel P, Tai E (2011). Melanoma in adolescents and young adults (ages 15-39 years): United States, 1999-2006. J Am Acad Dermatol.

[50] Liu X, Pang J, Xu F, Zhang X (2016). Simple approach to synthesize amino-functionalized carbon dots by carbonization of chitosan. Sci Rep.

[51] Sui X, Luo C, Wang C, Zhang F, Zhang J, Guo S (2016). Graphene quantum dots enhance anticancer activity of cisplatin via increasing its cellular and nuclear uptake. Nanomed Nanotechnol Biol Med.

[52] Smyder JA, Krauss TD (2011). Coming attractions for semiconductor quantum dots. Mater Today (Kidlington).

[53] Lee EY, Bae HC, Lee H, Jang Y, Park YH, Kim JH (2017). Intracellular ROS levels determine the apoptotic potential of keratinocyte by quantum dot via blockade of AKT phosphorylation. Exp Dermatol.

[54] Song J, Lin C, Yang X, Xie Y, Hu P, Li H (2019). Mitochondrial targeting nanodrugs self-assembled from 9-O-octadecyl substituted berberine derivative for cancer treatment by inducing mitochondrial apoptosis pathways. J Control Release.

[55] Ma Z-J, Lu L, Yang J-J, Wang X-X, Su G, Wang Z-l (2018). Lariciresinol induces apoptosis in HepG2 cells via mitochondrial-mediated apoptosis pathway. Eur J Pharmacol.

[56] Nguyen KC, Willmore WG, Tayabali AF (2013). Cadmium telluride quantum dots cause oxidative stress leading to extrinsic and intrinsic apoptosis in hepatocellular carcinoma HepG2 cells. Toxicology.

[57] Zhou J, Zhang L, Wang M, Zhou L, Feng X, Yu L (2019). CPX targeting DJ-1 triggers ROS-induced cell death and protective autophasy in colerecatal cancer. Theranostics.

[58] Chen Z, Liu X, Ma S (2016). The roles of mitochondria in autophagic cell death. Cancer Biother Radiopharm.

[59] Zhang J, Yu S-H (2016). Carbon dots: large-scale synthesis, sensing and bioimaging. Materials Today.

[60] Dasgupta A, Biancacci I, Kiessling F, Lammers T (2020). Imaging-assisted anticancer nanotherapy. Theranostics.

[61] Jiang X, Zong S, Chen C, Zhang Y, Wang Z, Cui Y (2018). Gold-carbon dots for the intracellular imaging of cancer-derived exosomes. Nanotechnology.

