# Supplementary material for: Multifunctional N-P-doped carbon dots for regulation of apoptosis and autophagy in B16F10 melanoma cancer cells and in vitro imaging applications
Source: Theranostics. 2020 Jun 22;10(17):7841–56. doi: 10.7150/thno.42291 (PMC7359102; doi:10.7150/thno.42291)
Supplement: Supplementary file 1 — Supplementary figures and tables. [file thnov10p7841s1.pdf]

Supplementary Information

**Multifunctional N-P-doped carbon dots for regulation of apoptosis and autophagy in B16F10 melanoma cancer cells and *in vitro* imaging applications**

Vivek K. Bajpai[1], Imran Khan[2,3], Shruti Shukla[4], Sung-Min Kang[5], Faisal Aziz[3], Kumud Malika Tripathi[6], Deepika Saini[7], Hye-Jin Cho[8], Nam Su Heo[9], Sumit K. Sonkar[7,*], Lei Chen[10,*], Yun Suk Huh[2,*], Young Kyu Han[1,*]

[1] Department of Energy and Materials Engineering, Dongguk University-Seoul, 30 Pildong-ro 1-gil, Seoul 04620, Republic of Korea

[2] Department of Biological Engineering, Biohybrid Systems Research Center (BSRC), Inha University, 100 Inha-ro, Nam-gu, Incheon 22212, Republic of Korea

[3] The Hormel Institute, University of Minnesota, Austin, MN, 55912, USA

[4] Department of Food Science and Technology, National Institute of Food Technology Entrepreneurship and Management (NIFTEM), Sonipat, Haryana 131028, India

[5] Wallace H. Coulter Department of Biomedical Engineering, Georgia Institute of Technology and Emory School of Medicine, Atlanta, Georgia, 30332, USA

[6] Department of Chemistry, Indian Institute of Petroleum and Energy, Visakhapatnam 531035, Andhra Pradesh, India.

[7] Department of Chemistry, Malaviya National Institute of Technology, Jaipur, Jaipur 302017, India

[8] Reliability Assessment Center for Chemical Materials, Korea Research Institute of Chemical Technology (KRICT), 141 Gajeong-ro, Yuseong-gu, Daejeon 305-600,

Republic of Korea

[9] Research Center for Materials Analysis, Korea Basic Science Institute (KBSI), Daejeon 34133, Republic of Korea

[10] College of Food Science, Fujian Agriculture and Forestry University, Fuzhou, Fujian 350002, China

**Running head:** Anticancer potential of N-P-doped carbon nanodots

**Corresponding authors: Sumit K. Sonkar** (E-mail: sksonkar.chy@mnit.ac.in); **Lei Chen** (E-mail: chenlei841114@hotmail.com); **Yun Suk Huh** (E-mail: yunsuk.huh@inha.ac.kr); **Young-Kyu Han (**E-mail: ykenergy@dongguk.edu)

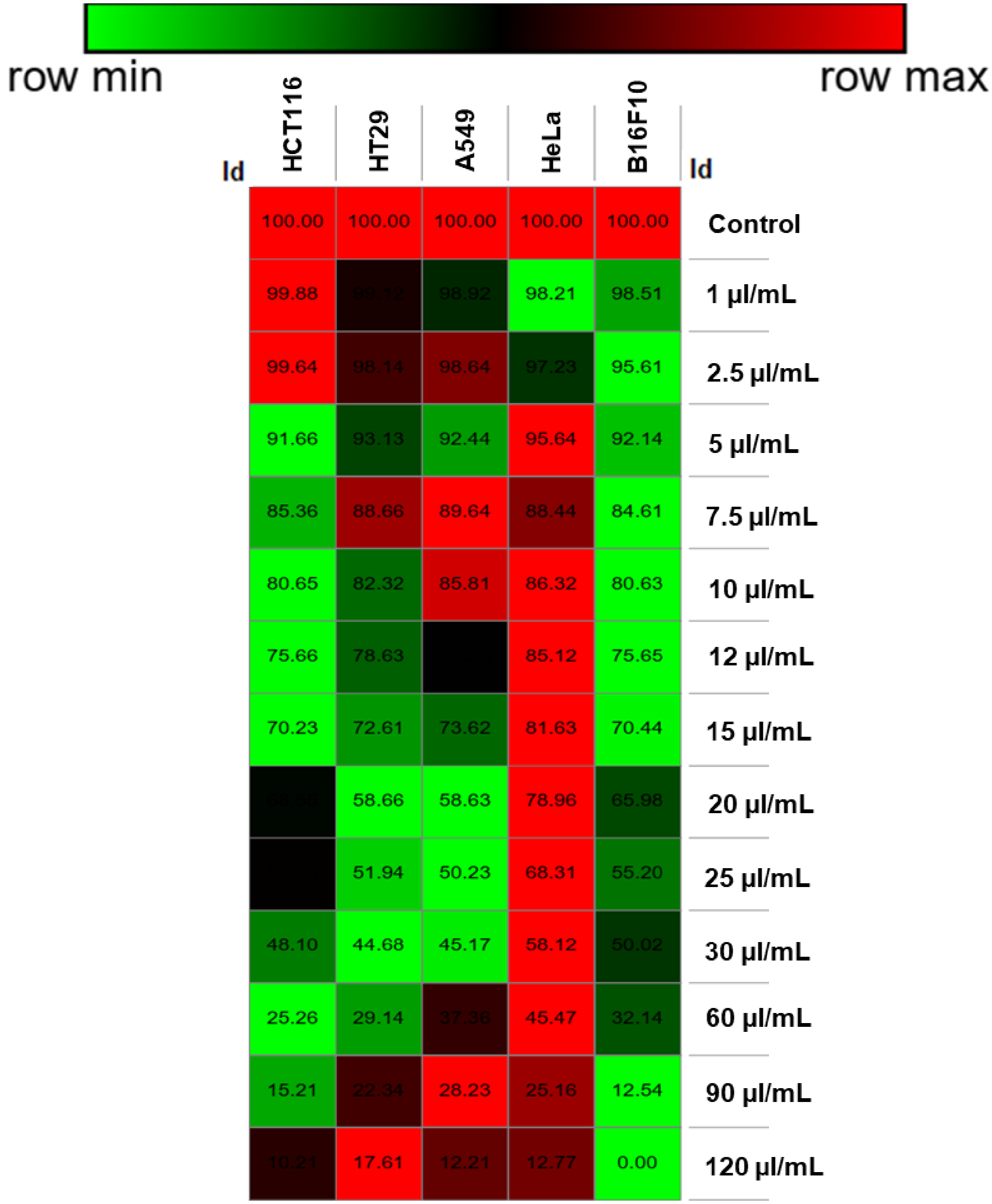


**Figure S1** Heatmap showing the anticancer activities (% cell viability) of NPCDs (0-120 µL/mL) on different cancer cells HCT116, HT29, A549, Hela, and B16F10 cells.

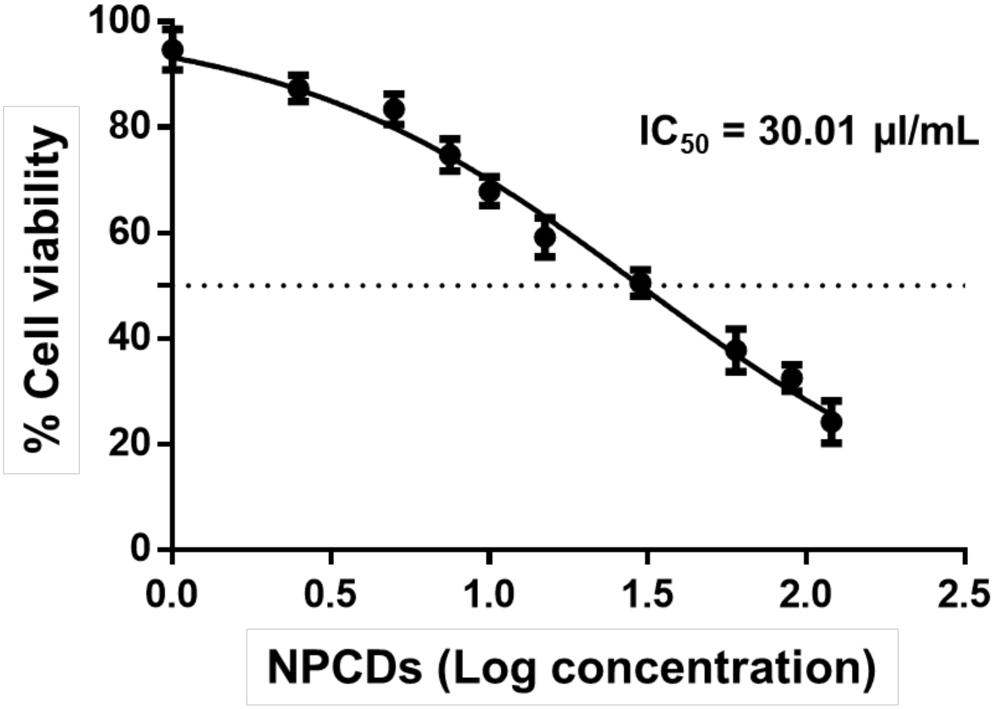


**Figure S2** Anticancer activity of NPCDs evaluated by MTT assay, effect of NPCDs (0-120 µL/mL) on B16F10 melanoma cells.

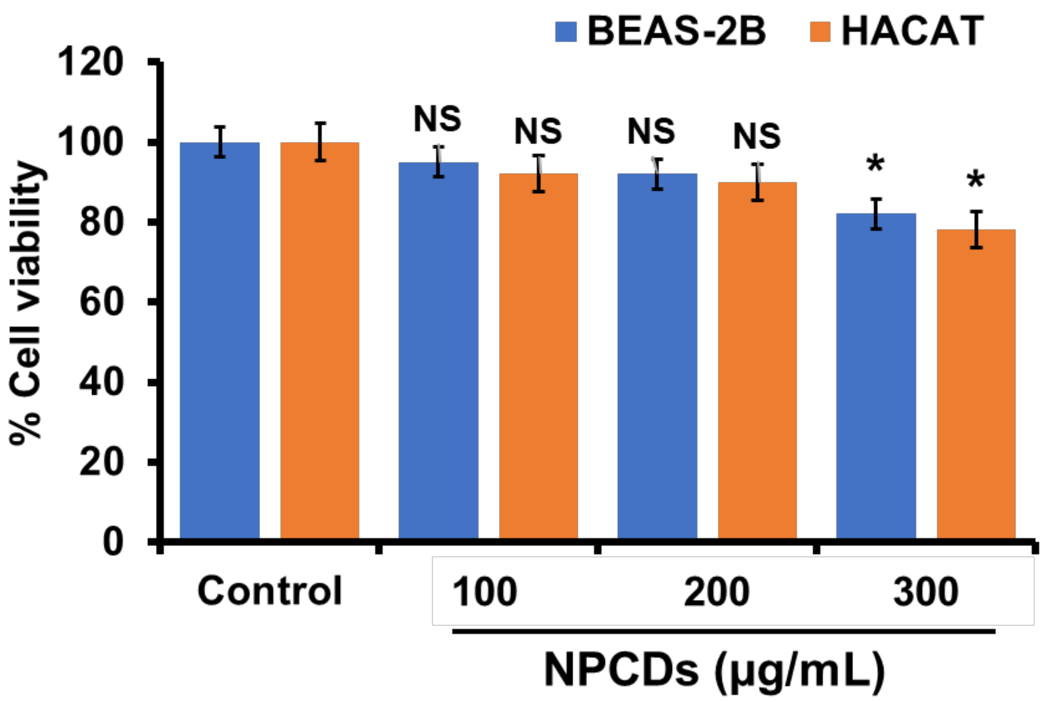


**Figure S3** Cytotoxicity of NPCDs on BEAS-2B human lung epithelial cells and HACAT as human keratinocytes.

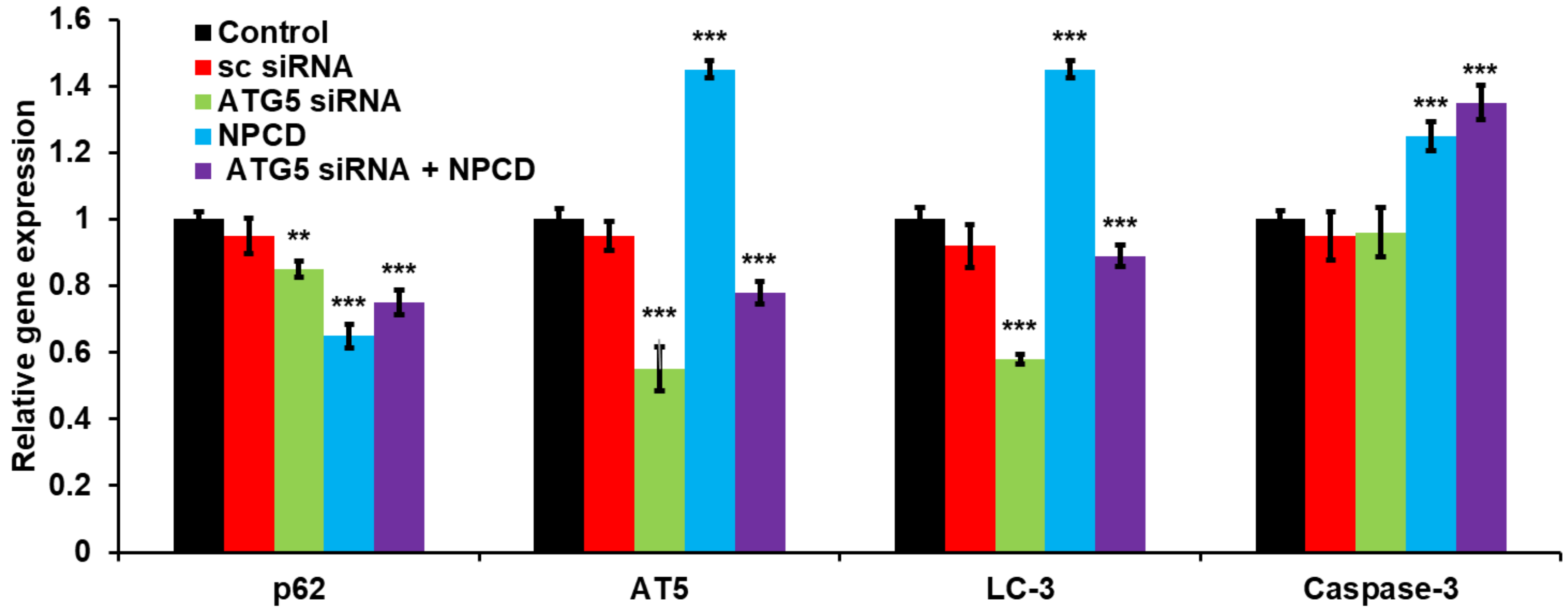


**Figure S4.** Autophagy attenuated by siRNA ATG5. The efficiency of siRNA mediated knockdown of ATG5 in B16F10 cells was examined by RT-PCR.

**Table S1** Primary and secondary antibodies used in the study.

| Sr. No. | Primary Antibody used | Dilution used | Secondary antibody used | Company |
|---|---|---|---|---|
| 1 | Bax (Rabbit) | 1:1000 | Anti-rabbit IgG, HRP-linked | Cell signaling technology |
| 2 | Bcl2 (Rabbit) | 1:1000 | Anti-rabbit IgG, HRP-linked | Cell signaling technology |
| 3 | Caspase-3 (Rabbit) | 1:1000 | Anti-rabbit IgG, HRP-linked | Cell signaling technology |
| 4 | β-actin (Rabbit) | 1:1000 | Anti-rabbit IgG, HRP-linked | Cell signaling technology |
| 5 | CDK-2 (Rabbit) | 1:1000 | Anti-rabbit IgG, HRP-linked | Cell signaling technology |
| 6 | CDK-4 (Rabbit) | 1:1000 | Anti-rabbit IgG, HRP-linked | Cell signaling technology |
| 7 | CDK-6 (Rabbit) | 1:1000 | Anti-rabbit IgG, HRP-linked | Cell signaling technology |
| 8 | p21 (Rabbit) | 1:1000 | Anti-rabbit IgG, HRP-linked | Cell signaling technology |
| 9 | LC-3 (Rabbit) | 1:1000 | Anti-rabbit IgG, HRP-linked | Cell signaling technology |
| 10 | p62 (Rabbit) | 1:1000 | Anti-rabbit IgG, HRP-linked | Cell signaling technology |
| 11 | ATG-5 (Rabbit) | 1:1000 | Anti-rabbit IgG, HRP-linked | Cell signaling technology |

Secondary antibody dilution used 1:10,000 (Cell Signaling Technology).

Restore™ PLUS Western Blot Stripping Buffer.

**Table S2** Primers used in this study.

| Primers | Sequence | Applications |
|---|---|---|
| *atg5-f* | AGGCAACCTGACCAGAAACA | Real time PCR |
| *atg5-r* | GAGGAAAGCAGAGGTGATGC | Real time PCR |
| *p62-f* | TGCCCAGACTACGACTTGTG | Real time PCR |
| *p62-r* | AGTGTCCGTGTTTCACCTTCC | Real time PCR |
| *lc3-f* | AGCAGCATCCAACCAAAATC | Real time PCR |
| *lc3-r* | CTGTGTCCGTTCACCAACAG | Real time PCR |
| *caspase3-f* | TGAGCCATGGTGAAGAAGGA | Real time PCR |
| *caspase3-r* | TCGGCCTCCACTGGTATTTT | Real time PCR |
| *βactin-f* | TCCCTGTATGCCTCTGGTCGT | Real time PCR |
| *βactin-r* | AAGCTGTAGCCTCTCTCGGTC | Real time PCR |
